# A scoping review about smoking, smoking cessation and their effects on anti-tuberculosis agents: insights into drug metabolisms, safety, and effectiveness

**DOI:** 10.3389/fphar.2025.1606150

**Published:** 2025-07-16

**Authors:** Carlo Maria Bellanca, Simone Pietro Polosa, Egle Augello, Giulia Di Benedetto, Chiara Burgaletto, Anna Flavia Cantone, Gabriella Gaudio, Giuseppe Nunnari, Davide Campagna, Jennifer M. Nailes, Hamza Shahbaz, Reza Kurniawan Tanuwihardja, Anant Mohan, Manuela Ceccarelli, Renato Bernardini, Andrea Marino, Giuseppina Cantarella

**Affiliations:** ^1^ Department of Biomedical and Biotechnological Sciences, Section of Pharmacology, University of Catania, Catania, Italy; ^2^ Clinical Toxicology Unit, University Hospital of Catania, Catania, Italy; ^3^ Department of Biomedical Sciences, Section of Neuroscience and Clinical Pharmacology, University of Cagliari, Cagliari, Italy; ^4^ Department of Biochemical Engineering, University College London, London, United Kingdom; ^5^ Unit of Infectious Diseases, Department of Clinical and Experimental Medicine, University of Catania, ARNAS Garibaldi Hospital of Catania, Catania, Italy; ^6^ Center of Excellence for the Acceleration of HArm Reduction (CoEHAR), University of Catania, Catania, Italy; ^7^ Unitá Operativa Complessa (UOC) Medicina e Chirurgia d'Accettazione e Urgenza (MCAU), University Teaching Hospital “Policlinico-Vittorio Emanuele”, University of Catania, Catania, Italy; ^8^ Department of Clinical and Experimental Medicine, University of Catania, Catania, Italy; ^9^ Department of Preventive and Community Medicine, University of The East Ramon Magsaysay Memorial Medical Centre, Quezon City, Philippines; ^10^ Pulmonology Department, Jinnah Hospital, Lahore, Pakistan; ^11^ Department of Pulmonology, Allama Iqbal Medical College, Lahore, Pakistan; ^12^ Departemen Pulmonologi dan Kedokteran Respirasi, Dr. H.A. Rotinsulu Lung Hospital, Bandung, Indonesia; ^13^ Department of Pulmonary Medicine, All India Institute of Medical Sciences, New Delhi, India; ^14^ Unit of Infectious Diseases, Department of Medicine and Surgery, “Kore” University of Enna, Enna, Italy

**Keywords:** cytochrome P450, drug-drug interactions, adverse drug reactions, antibiotics, nicotine, smoking cessation

## Abstract

The World Health Organization (WHO) ranks tuberculosis (TB) as one of the top 10 causes of deaths worldwide. Notably, tobacco smoking represents a significant promoting factor in TB progression, being associated with poorer treatment outcomes, delayed conversion to negative smear or culture, and higher dropout rates from treatment plans. Remarkably, high rates of smoking and TB frequently overlaps in the same countries, warranting the need for targeted public health interventions. Prioritising smoking cessation is essential for smokers with TB, as sustained abstinence has been associated with reduced mortality and a more successful cure. This review examines the intricate relationship between cigarette smoking, smoking cessation therapies and anti-TB drugs, focusing on the impact of tobacco smoking compounds on liver detoxifying systems, such as influence of polycyclic aromatic hydrocarbons (PAHs) on hepatic cytochrome P450 (CYP450) enzymes mostly, and on metabolism of antituberculous medications. Integrating smoking cessation and TB treatment programmes must also take into account potential drug-drug interactions between smoking cessation medications and anti-TB drugs, a critical area for patient safety and effective TB management. This review article aims to provide healthcare professionals with the knowledge to better support TB patients who smoke or are intending to quit, to ensure tailored and effective treatment strategies, while highlighting gaps in current research and advocating for further studies to fill these gaps.

## Graphical Abstract

**Figure:**
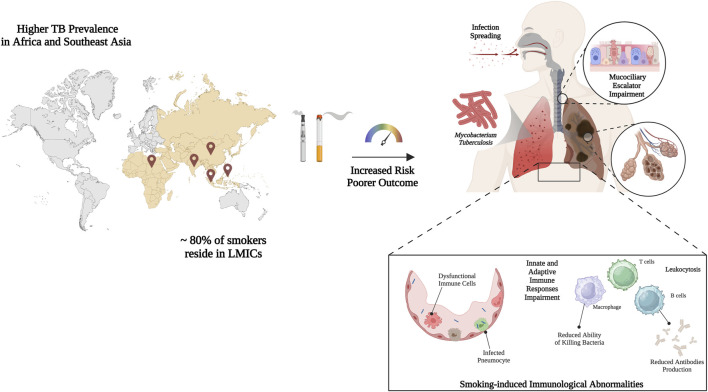
Graphic representation of the overlap between smoking and tuberculosis prevalence worldwide, especially in low-middle income countries (LMICs), which highlights the existing relationship and the impact of smoking habit in worsening disease outcome. Smoking increases infection odds and accelerates disease course altering immunological parameters and escalating histopathological damages. More specifically, it slows mucociliary clearance, injures the epithelial barrier, and depletes surfactant proteins, facilitating bacillary entry into the alveoli. Created with BioRender.com; accessed on 12 January 2025.

## 1 Introduction

Tuberculosis (TB) is a preventable, treatable, and curable infectious disease that primarily affects the lungs. Despite advances in medical treatment, TB remains a significant global health challenge (Bloom et al., 2017). In 2022, an estimated 10.6 million people developed TB, with a high prevalence in South-East Asia (notably India, Indonesia, China, the Philippines, Pakistan, and Bangladesh) and Africa (including Nigeria and the Democratic Republic of the Congo). Additionally, approximately 1.3 million people were estimated to have died from TB that same year. Thus, the World Health Organization (WHO) ranks TB as a leading killer infectious disease and one of the top 10 causes of deaths worldwide (Global Tuberculosis Report, 2023).

In Europe, TB incidence has generally decreased over the past decade, with the European Centre for Disease Prevention and Control (ECDC) reporting an average rate of around eight cases per 100,000 people in the European Union (EU)/European Economic Area (EEA) in 2022, with a total of 36,179 TB cases. Eastern European countries such as Romania and Lithuania still face higher TB rates, contributing to the continent’s overall case burden. In Italy, the incidence of TB is relatively low compared to global rates, with around 4.1 cases per 100,000 people in 2022, with 2,439 new cases. However, certain high-risk populations, including recent immigrants, individuals with HIV, and vulnerable urban populations, still experience a notable risk of TB (TB Incidence, 2024).

The susceptibility of these groups is multifactorial. Immigrants often arrive from countries with high TB endemicity and may experience reactivation of latent TB due to stress, malnutrition, or crowded living conditions in the host country (Hayward et al., 2018; Pareek et al., 2016). People living with HIV (PLHIV) are particularly susceptible because of a compromised immune system, as the depletion of CD4^+^ T-cells critically impairs the body’s ability to control *Mycobacterium tuberculosis* (Mtb) infection, leading to a significantly higher risk of progression from latent to active disease (Getahun et al., 2010). The WHO estimates that PLHIV are 18 times more likely to develop active TB than HIV-negative individuals (Tuberculosis and HIV, 2025; TB Preventive Treatment, 2025). Vulnerable urban populations, including homeless and those residing in low-income settings, are exposed to a potent mix of biological insults due to factors like poor ventilation in shelters, household crowding, and limited access to healthcare, all of which facilitate disease transmission. A systematic review and meta-analysis investigating the risk of TB among populations living in slum settings, reported an odds ratio of 2.96 (2.84–3.09) for smear-positive TB among dwellers compared with national averages (Noykhovich, Mookherji, and Roess, 2019).

Taken together these data show that each population’s elevated TB burden results from an interplay of biological susceptibility and adverse social conditions. Effective TB control must therefore couple biomedical interventions–systematic Latent Tuberculosis Infection (LTBI) screening of recent migrants, integrated TB–HIV services, smoke-free housing initiatives–with policies that tackle the underlying social determinants of health, such as overcrowding, insecure employment, and inadequate access to care (Zumla et al., 2025; Feldman et al., 2024; Lönnroth et al., 2009).

One significant factor promoting TB disease progression is smoking (Amere et al., 2018). Currently, 1.3 billion people worldwide use tobacco, primarily through smoking, which leads to over seven million deaths annually from smoking-related illnesses (Tobacco Collaborators, 2017 Tobacco Collaborators, 2017; Tobacco Collaborators, 2021 Tobacco Collaborators, 2021; WHO Report on the Global Tobacco Epidemic, 2023).Tobacco smoking is a major risk factor noy only for lung cancer, chronic obstructive pulmonary disease (COPD), and cardiovascular diseases, but also significantly contributes to the global burden of TB, particularly in low- and middle-income countries (LMICs) (Gajalakshmi et al., 2003; Song et al., 2024; Dana et al., 2024; Riccardi et al., 2024; Pampaloni et al., 2021).

Remarkably, high rates of smoking and TB frequently overlaps in the same countries (Global Tuberculosis Report, 2023; WHO Report on the Global Tobacco Epidemic, 2023; TB Disease Burden, 2024; WHO Global, 2025). For instance, countries with a high TB burden such as India, Indonesia, China, the Philippines, and Bangladesh also report high smoking prevalence. This overlap is not coincidental but is rooted in shared socioeconomic determinants. Factors such as poverty, crowded living conditions, limited access to education and healthcare, and malnutrition create a fertile ground for both TB transmission and the adoption of smoking behaviours. Studies have shown a direct correlation between smoking prevalence and TB incidence at a population level, suggesting that tobacco use acts as a significant driver of the TB epidemic in these regions, contributing to increased transmission, disease progression, and mortality (WHO Global, 2024; Jha et al., 2008).

These data suggest that the geographic overlap of high TB burden and intense tobacco use is more than accidental. Indeed, tobacco smoke is likely to amplify TB risk through both direct biological effects (immune suppression, altered drug metabolism) and shared socio-economic determinants.

Approximately 80% of the world’s smokers live in LMICs, where the majority of TB deaths occur. It is estimated that 20% of TB cases are associated with smoking (National Center for Chronic Disease Prevention and Health Promotion Office on Smoking and Health, 2014).

This co-occurrence presents significant public health challenges, as tobacco use is detrimental to TB. There is sufficient evidence to infer a causal relationship between smoking and increased risk of TB disease, recurrent disease, and mortality (National Center for Chronic Disease Prevention and Health Promotion Office on Smoking and Health, 2014). Smokers have a TB disease risk approximately twice that of their non-smoking counterparts. It is also possible that smoking impacts the progression to active disease. A recent meta-analysis has shown that smoking is associated with poorer treatment outcomes, delayed conversion to negative smear or culture, and higher dropout rates from treatment plans, ultimately leading to a lower likelihood of treatment success (E. Y. Wang et al., 2020).

Stopping smoking may have significant benefits for individuals with TB. Indeed, successful abstinence from smoking in TB care has been shown to lead to better treatment outcomes for TB patients, including a substantially lower rate of treatment default and failure, better sputum conversion rate, and improved radiological findings (Awaisu et al., 2011). Therefore, prioritizing smoking cessation is vital for smokers with TB. The WHO underscores the significance of smoking cessation for patients with TB (A Guide for Tuberculosis Patients to Quit Smoking, 2025).

When discussing smoking cessation in individuals with TB, it is crucial to acknowledge that the substances in tobacco smoke can alter various liver enzyme detoxification pathways. These alterations can significantly impact the pharmacokinetics of certain antituberculous medications. On the other hand, smoking cessation can lead to a gradual normalization of these metabolic pathways, similarly affecting the pharmacokinetics of drugs. Consequently, during the quitting process, it may be necessary to adjust or lower the dosages of some medications to ensure both effective and safer treatment.

This review article aims to explore the interrelationship between smoking, the process of smoking cessation, and the treatment of TB. An evaluation of the impact of smoking on the effectiveness and metabolism of antituberculosis medications is also conducted. Furthermore, it delves into the potential interactions between pharmaceutical agents employed in smoking cessation programmes and antitubercular treatments. By highlighting these interactions, this review could offer valuable insights for healthcare providers to enhance treatment approaches for TB patients who smoke or have recently stopped, and for researchers to identify potential areas for further study.

## 2 Methods

A comprehensive review of the literature was conducted from July 2024 to October 2024, encompassing a wide range of research methodologies, including observational studies, randomised and non-randomised clinical trials, experimental studies, case reports, and case series. The primary objective of this review was to identify studies that offered valuable insights into the potential metabolic interactions between smoking, smoking cessation, and smoking cessation medications, with a focus on their impact on the pharmacokinetics and pharmacodynamics of antituberculous drugs. The search was performed in both MEDLINE (PubMed) and EMBASE. Customised search strings were created and employed to locate relevant studies, the details of which are provided in Supplementary Material. Additional specialized databases like DrugBank.com, Knox et al. (2024), PharmGKB, (2024), along with the Summary of Product Characteristics (SmPC), were also examined for each medication. The titles and abstracts of these articles were then reviewed by CMB, SPP, EA and AM to identify studies for a comprehensive evaluation. Studies that appeared suitable for inclusion or could not be definitively excluded based solely on the title and abstract were advanced for further assessment. The Zotero software (Zotero 6.0.30; Roy Rosenzweig Centre for History and New Media (RRCHNM); George Mason University) was employed for the management of records and the elimination of duplicates. Following this, the remaining full-text articles were independently evaluated by other authors, with any disagreements resolved through discussion and consensus. Articles that met the established inclusion criteria were selected for the qualitative synthesis. Additionally, the references cited in the included articles and review papers were further examined to discover any potentially relevant studies. A standardized data extraction form specifically created for this purpose was utilised to gather pertinent data. The extracted details, such as authors, study population, study design, definition of smoking status, median follow-up time and outcomes, were systematically documented and organised in a table. The qualitative synthesis focused on the clinical significance of smoking cessation in relation to TB-related complications.

This review consolidates the evidence on the metabolic and clinical relationships between smoking and TB. It highlights how smoking affects both disease progression and the pharmacokinetics of treatments. The paucity of data on specific enzyme pathways and differences in study populations may affect the applicability of the findings. In addition, the review focuses primarily on pharmacological issues, with less emphasis on behavioural and socioeconomic elements.

## 3 Impact of tobacco smoke on enzymatic drug metabolism

Tobacco smoke is a complex mixture of polycyclic aromatic hydrocarbons (PAHs), ammonia, aromatic amines, phenols, carbonyls, hydrocyanic acid, and N-nitrosamines (Hoffmann and Hoffmann, 1997). Numerous of these chemicals interact with the enzymes involved in xenobiotic metabolism and various transporters, thus affecting the biotransformation of substances. Therefore, cigarette smoking, as well as quitting, may impact drug metabolism (Maideen, 2019).

Particularly, PAHs formed during incomplete tobacco combustion influence the hepatic cytochrome P450 (CYP450) enzyme system, especially by induction of isoenzymes CYP1A1, 1A2, 1B1, and 2E1, leading to accelerated drug clearance, potentially lowering blood concentrations. As a consequence, therapeutic monitoring and eventually dose adjustment would be required (Stepanov et al., 2010; McDonnell and Dang, 2013; Meech and Mackenzie, 1997). Moreover, mounting evidence points to the possibility that enzyme function may undergo alterations, potentially attributable to epigenetic processes, which could in turn result in a sustained increase in metabolic rate even after the cessation of smoking (Hirota et al., 2008; O’Malley et al., 2014).

Among the isoenzymes, the induction of CYP1A2 is of clinical importance because many drugs are its substrates. A study found a 1.66-fold increase in CYP1A2 activity in smokers of 11–20 cigarettes per day, which is reversed after smoking cessation (Tantcheva-Poór et al., 1999). Within 4 days of quitting, caffeine clearance, a measure of CYP1A2 activity, fell by 36% (Faber and Uwe, 2004). For former heavy smokers taking CYP1A2-metabolized drugs, dose adjustments after cessation are essential to avoid elevated drug levels and adverse drug reactions (ADRs). CYP1A1 metabolic activity has also been shown to be increased by approximately 66%–70% in smokers, mainly due to exposure to aryl hydrocarbons such as benzo [a]pyrene (Vistisen, Loft, and Poulsen, 1991). It also appeared that tobacco use affects the quantitative mRNA expression of CYP1B1, the induction of which may be influenced by genetic polymorphisms (Helmig et al., 2010).

The enhanced CYP2E1 expression and activity (Villard et al., 1998; Seree et al., 1996) in smokers has been demonstrated by accelerated metabolism of substances such as chlorzoxazone of about 24% (Benowitz et al., 2003).

Conversely, the impact of PAHs on uridine diphosphate (UDP)-glucuronosyltransferases (UGTs) appears to be complex and unclear, with a paucity of research in this area. However, given the inconsistency and heterogeneity of findings, it can be inferred that the effects may vary between different UGT isoforms (Collier et al., 2002; Court, 2010; Dragacci et al., 1987).

Nicotine, a well-known component of cigarette smoke, also alter metabolic pathways particularly by interfering with Organic Cation Transporters (OCTs) *in vitro* (Bergen et al., 2014; Urakami et al., 1998; Lips et al., 2005). Its impact on CYP2E1, CYP2A1/2A2, and CYP2B1/2B2 has been demonstrated within the central nervous system (Anandatheerthavarada et al., 1993a; Anandatheerthavarada et al., 1993b), though the clinical significance remains unclear (Zevin and Benowitz, 1999). While pharmacokinetics interactions are mainly attributable to PAHs, nicotine does alter and possibly negate drug effects by activating the sympathetic nervous system (N. L. Benowitz, 1997). Additionally, it has been reported that nicotine metabolism is impaired and even reduced by tobacco smoke through the inhibition of CYP2A6, which is mainly responsible for the conversion to cotinine (Lee et al., 1987; Benowitz and Jacob, 1993; 2000). Alongside PAHs and nicotine, other smoke constituents like acetone and carbon monoxide (CO) may affect hepatic enzymes but are considered less impactful (Zevin and Benowitz, 1999; Bellanca et al., 2024).

Beyond the impact on drug-metabolising enzymes, components of tobacco smoke exert various effects at cellular and subcellular level. Many studies have examined the influence of individual components, such as nicotine, aryl hydrocarbon, acetylcholine, and acrolein but the biological consequences stem from prolonged exposure to all components combined.

Cigarette smoke profoundly undermines the host’s capacity to contain Mtb at every stage of infection. In the airways it slows mucociliary clearance, injures the epithelial barrier, and depletes surfactant proteins, facilitating bacillary entry into the alveoli (Tilley et al., 2015; Moré et al., 2010; Honda et al., 1996; Corleis et al., 2023). Once there, smoke-expanded pools of alveolar macrophages are paradoxically less effective: phagocytosis, autophagy, antigen presentation, and production of key cytokines such as tumor necrosis factor-α (TNF-α), interleukin-12 (IL-12), and interferon-γ (IFN-γ) are all attenuated, while a shift toward anti-inflammatory M2 phenotypes and smoke-induced metabolic exhaustion enable intracellular survival of the pathogen (Leemans et al., 2005; Hodge et al., 2007; O’Leary et al., 2014; Chen et al., 2007; Smit et al., 2014).

The innate immune response is further compromised by an influx of hypo-functional neutrophils. Defective efferocytosis leaves bacteria-laden dying cells to rupture, thereby amplifying tissue damage and bacillary spread (Dallenga et al., 2017; Kirkham et al., 2004). Down-stream, dendritic cells exposed to smoke migrate poorly to lymph nodes and bias T-cell priming away from protective Th1 immunity, favouring Th2, Th17, and regulatory T-cell profiles marked by exhaustion receptors such as PD-1 and CTLA-4 (Feng et al., 2011; McNab et al., 2011; Quan et al., 2022).

Emerging evidence indicates that smoking induces gut dysbiosis, altering the composition and function of intestinal bacteria. Since the microbiome contributes to systemic immunity and can metabolise certain drugs, this may represent an indirect mechanism affecting both host susceptibility to TB and the bioavailability of orally administered anti-TB agents (Fan et al., 2023; Leite et al., 2022; Belkaid and Hand, 2014; Zhao et al., 2023).

At a subcellular level, toxins in cigarette smoke, such as cadmium and acrolein, are known to induce mitochondrial dysfunction and increase oxidative stress. This can impair cellular energy production and trigger apoptotic pathways in immune cells, further weakening the host response. Moreover, the added oxidative burden could potentially exacerbate drug-induced toxicities (Zhang et al., 2024; Wang et al., 2017; Aridgides et al., 2019; Lugg et al., 2022).

## 4 Effects of tobacco smoke on metabolism of antituberculous medications

Therapeutic options for TB encompass both first- and second-line drugs. The current standard for drug-sensitive TB (DS-TB) is a 6-month regimen comprising isoniazid (INH), rifampin (also known as rifampicin) (RIF), pyrazinamide (PZA), and ethambutol (EMB), starting with a 2-month intensive phase of all four drugs, followed by 4 months of INH and RIF (Alsayed and Gunosewoyo, 2023). However, as a result of the rise in antibiotic resistance, second-line drugs are now essential (Drug-Resistant Tuberculosis, 2024). Drug-resistant TB (DR-TB) is defined by resistance to at least one of the first-line drug, while multidrug-resistant TB (MDR-TB) implies resistance to both RIF and INH, and extensively drug-resistant TB (XDR-TB) refers to MDR-TB strains that are resistant to fluoroquinolones (FQ) and at least one aminoglycoside (Johnson et al., 2024). Recent advances, primarily based on three landmark clinical studies, namely, Nix-TB, ZeNix, and TB PRACTECAL (Conradie et al., 2020; 2022; Nyang’wa et al., 2022), led to the introduction of 6–9 months regimens of bedaquiline, pretomanid, linezolid, with or without moxifloxacin (BPaL-M or BPaL, respectively), offering improved outcomes compared to older treatment strategies (Johnson et al., 2024).

On this ground the WHO updated its DR-TB treatment guidelines in 2022 (WHO Consolidated Guidelines on Tuberculosis, 2024), recommending the 6-month BPaL-M regimen for MDR/Rifampicin-Resistant (RR)-TB in patients unexposed to bedaquiline, pretomanid, or linezolid (Johnson et al., 2024). For patients diagnosed with FQ-susceptible strains of Mtb, a 9-month all-oral regimen is advised, while those with FQ resistance may receive BPaL without moxifloxacin. Longer regimens remain viable for patients with additional resistance, intolerance to short-course drugs, severe disease, pregnancy, certain extrapulmonary TB cases, or other complex needs, with alternative agents recommended as needed (i.e., streptomycin, levofloxacin, clofazimine, and delamanid) (Vanino et al., 2023).

There is compelling evidence of an association between TB and smoking habits, in terms of increased risk of poorer outcome and defaulting on antituberculosis treatment (Bates et al., 2007; Batista et al., 2008). Furthermore, there is evidence that tobacco-induced immunological changes are reversible 6 months after smoking cessation (Arcavi and Benowitz, 2004; Miller et al., 1982; Hughes et al., 1985; Sopori, 2002). Therefore, integrating treatment regimens to eradicate Mtb infection and smoking cessation therapies appears to be an effective strategy to act on both fronts and ultimately lead to improved health and reduced healthcare burden (Aryanpur et al., 2016; Novotny, 2008; Schneider and Thomas, 2007). However, one of the major problems is the plausible occurrence of drug-drug interactions (DDIs), as both pathological conditions require long-term medical therapies. Indeed, the shortest treatment regimens for TB last at least 6 months, making the occurrence of DDIs not only likely but probable. Further widening the population at risk of DDIs is the need to provide TB preventive treatment (TPT) to all those at high risk of contracting the disease. Indeed, regimens based on RIF, INH or levofloxacin are recommended for HIV positive patients, cohabitants of affected persons, healthcare workers, and other categories of people engaged in high TB prevalence environments (WHO Consolidated Guidelines on Tuberculosis Module 1, 2024; Tanoglu et al., 2023).

As previously discussed, tobacco smoke is a mixture of substances exhibiting varying abilities to induce or inhibit the activity of hepatic detoxifying enzymes, as well as other transporters involved in the elimination of xenobiotics. In addition, the reversible nature of enzyme induction after smoking cessation emphasizes the need for careful management and dose adjustments of medications in quitters to ensure optimal pharmacotherapy. A further complicating factor is the extensive range of chemotherapeutic antibiotics that are available for the treatment of TB, each of which possesses its own distinctive pharmacokinetic and pharmacodynamic characteristics. It is therefore crucial to ascertain whether the patient is a smoker, bearing in mind the substantial degree of overlap between the two conditions, and whether they are undergoing smoking cessation therapy or intending to start it while simultaneously affected by TB. In this context, it is of utmost importance to analyse the metabolism of individual molecules with a view to establishing a highly personalised therapy.

INH is a prodrug that enters the Mtb cytoplasm via passive diffusion, where it inhibits mycolic acid synthesis by targeting the InhA enzyme, an essential catalyst in an early step of the mycolic acid biosynthetic pathway (Bardou et al., 1998; Mitchison, 1956). INH initially exerts a bacteriostatic effect for the first 24 h of treatment, followed by a bactericidal activity against proliferating Mtb at therapeutic levels (Unissa et al., 2016).

INH is mainly metabolized in the liver through two major pathways to metabolites such as acetyl isoniazid (AcINH), hydrazine (Hz), acetyl hydrazine (AcHz), diacetyl hydrazine (DiAcHz), and isonicotinic acid (INA), primarily mediated by arylamine N-acetyl transferase2 (NAT2) and amidases (Wang et al., 2016; Ellard and Gammon, 1976; McKENNIS et al., 1959). Further oxidation of Hz and AcHz by CYP450 enzymes, particularly CYP2E1, generates reactive intermediates that form covalent adducts with endogenous macromolecules, a process implicated in INH-induced hepatotoxicity (Delaney and Timbrell, 1995; Yue et al., 2004; Metushi and Jack, 2014).

The rifamycins are a family of antibiotics including rifampin, rifabutin, and rifapentine, which bind to the ß-subunit of bacterial DNA-dependent RNA polymerase (RNAP), inhibiting RNA synthesis and causing Mtb death (Abulfathi et al., 2019). Rifamycins are well-absorbed orally, with RIF showing high bioavailability, although food can reduce its absorption. They distribute widely throughout body tissues and fluids, reaching effective concentrations against intracellular pathogens. Specifically, RIF undergoes hepatic metabolism to its active metabolite, 25-desacetyl-rifampicin, and is a well-characterized inducer of multiple CYP450 enzymes, including CYP3A4, CYP1A2, CYP2B6, CYP2C8, CYP2C9, CYP2C19, and UGTs. It also affects transporters such as P-glycoprotein (P-gp) and multidrug resistance-associated protein-2 (MRP2), leading to significant DDIs risk. Enzyme induction shortens rifampin’s half-life with prolonged use, making daily dosing necessary. Excreted mainly in bile, with some renal elimination, it can turn body fluids orange-red, a harmless but noticeable ADRs. Hepatotoxicity remains a concern, thus liver function monitoring during therapy is essential (Jamis-Dow et al., 1997; Nakajima et al., 2011; Rae et al., 2001; Sumida et al., 2000; Dalet-Beluche et al., 1992; Niemi et al., 2003; Rifadin, 2023).

Rifabutin, a more hydrophobic rifamycin with reduced enzyme induction potential and fewer DDIs, is particularly valuable in Mtb/HIV coinfections. It also has a longer half-life, allowing less frequent dosing. Of its five identified metabolites, 25-O-desacetyl and 31-hydroxy are the most predominant (Knox et al., 2024; Crabol et al., 2016; Kim et al., 2022; Blaschke and Skinner, 1996). Rifabutin act as a weaker inducer than RIF, with 2–3 times lower effect on CYP3A4, making drug interactions less impactful in clinical practice (AIFA - Ricerca Farmaco, 2024).

Rifapentine, whose half-life is the longest, enables weekly administrations. It is known to induce CYP3A4 and CYP2C8/9 enzymes, starting within 4 days of the first dose and returning to baseline 14 days after discontinuation. Studies suggest that the induction potential of rifapentine is less than that of RIF but greater than that of rifabutin (Priftin, 2000).

PZA is a prodrug that converts to its active form, pyrazinoic acid (PA), under acidic conditions inside Mtb. PA then diffuses back into the bacilli, where it accumulates and exerts multiple effects against Mtb, including inhibition of fatty acid synthase, disruption of membrane potential, and interference with energy production by interacting with ribosomal protein S1 to inhibit trans-translation (Boshoff, Mizrahi, and Barry, 2002; Zimhony et al., 2007; Ngo et al., 2007; Shi et al., 2011).

PZA is primarily metabolized to PA in the liver by amidase. PA can be further oxidized by xanthine oxidase (XO) to form 5-hydroxy-pyrazinoic acid (5-OH-PA), a metabolite thought to be more hepatotoxic than PA. Alternatively, PZA can be initially oxidized to 5-hydroxy-pyrazinamide (5-OH-PZA) by XO, followed by amidase-mediated hydrolysis to 5-OH-PA. PZA and its metabolites are mainly excreted by the kidney (American Medical Association, 1992; Lacroix et al., 1989).

The hepatotoxicity of PZA is dose-dependent, especially at doses above 40 mg/kg, and correlates with its hepatic metabolism, suggesting a direct toxic effect rather than a hypersensitive or immune-mediated mechanism. Experimental studies in Wistar rats treated with PZA or PA showed hepatotoxicity, as demonstrated by elevated serum alanine aminotransferase, aspartate transaminase, and galactose single-point levels (Tostmann et al., 2008; Shih et al., 2013). Recent studies further confirmed 5-OH-PA as the most toxic metabolite, causing liver damage and metabolic shifts in rats (Rawat et al., 2018; Hussain, Zhu, and Ma, 2021).

EMB diffuses into Mtb cells and inhibits the arabinosyltransferases (embA, embB, and embC), thus disrupting the formation of cell wall components like arabinogalactan and lipoarabinomannan, ultimately hindering cell division (Myambutol, 2008; L. Zhang et al., 2020; Goude et al., 2009; Amin et al., 2008). Around 50% of an EMB dose is excreted unchanged in the urine, with an additional 8%–15% appearing as metabolites, and about 20%–22% is found unaltered in faeces. The main metabolic pathway involves oxidation by aldehyde dehydrogenase to an aldehyde metabolite, which is converted to the dicarboxylic acid 2,2'-(ethylenediimino)di-butyric acid (Myambutol, 2008; Peets et al., 1965).

Although EMB metabolism does not involve CYP450 enzymes, a study by Lee S.Y. et al. in 2014 found that it inhibits several CYP isoforms in human liver microsomes. Using liquid chromatography-electrospray ionization tandem mass spectrometry, EMB has been reported to inhibit CYP1A2 and CYP2E1 strongly, CYP2C19 and CYP2D6 moderately, and CYP2A6, CYP2C9, and CYP3A4 weakly (Lee et al., 2014).

Streptomycin, the first drug available for the treatment of Mtb, is now largely a second-line option due to concerns about resistance and toxicity (Waters and Tadi, 2024), indicated in MDR-TB and various non-tuberculosis infections. It is an aminoglycoside which exhibits bactericidal effects both by disrupting cell membranes and impairing protein synthesis through binding to the 16S rRNA in helix 44 (h44) near the A site of the 30S ribosomal subunit. This binding displaces residues A1492 and A1493 in h44, mimicking correct codon-anticodon pairing, which impedes translation and other steps in protein synthesis (Serio et al., 2018; Bulitta et al., 2015). Recent studies indicate that aminoglycosides bind to an additional cryptic site in the 23S rRNA of the 50S subunit, contributing to translation errors that destabilize membrane structure (Sullivan et al., 2018; Ying et al., 2019). Misincorporated proteins may integrate into the cell membrane, enhancing the bactericidal effect by further damaging bacterial integrity (Wallace et al., 1973; Borovinskaya et al., 2007).

Because of poor oral absorption, streptomycin is administered parenterally, typically by intramuscular injection, and less often intravenously. Peak serum concentrations (25–50 mcg/mL) are achieved within an hour after 1 g intramuscular dose (Waters and Tadi, 2024). No significant human metabolites of streptomycin have been identified, with 50%–60% of the drug excreted unchanged in the urine (Chandra Acharya and Kurosu, 2023).

Capreomycin, used primarily as a second-line treatment for MDR-TB, has an unclear mechanism of action. It is thought to inhibit protein synthesis by binding to the 70S ribosomal unit, thereby causing abnormal proteins essential for bacterial survival to be produced, ultimately leading to bacterial cell death (Knox et al., 2024; Capastat, 2019).

Capreomycin is administered parenterally, via intramuscular or intravenous injection, as it cannot be efficiently absorbed if taken orally (Capastat, 2019).

Amikacin is a semi-synthetic aminoglycoside derived from kanamycin A, given as a second-line treatment for MDR-TB and several Gram-negative bacterial infections. It interacts with the bacterial 30S ribosomal subunit, interfering with mRNA binding and tRNA acceptor sites. The disruption of protein synthesis results in the production of non-functional or toxic peptides, leading to bacterial cell death (Arikayce, 2023).

Amikacin exerts bactericidal effects against both Gram-positive and Gram-negative bacteria, including strains resistant to other aminoglycosides like gentamicin and tobramycin. It does not undergo appreciable metabolism, which enhances its stability against bacterial enzymatic deactivation, thereby reducing resistance occurrence (Knox et al., 2024; Arikayce, 2023).

Due to poor oral bioavailability, amikacin is administered parenterally. Roughly 50%–60% of an administered dose is excreted unchanged in the urine, making it mainly cleared through the kidneys (Chandra Acharya and Kurosu, 2023; Arikayce, 2023).

Levofloxacin is a fluoroquinolone antibiotic, specifically the S-(−) isomer of racemic ofloxacin. It inhibits the activity of two key bacterial enzymes, DNA gyrase and topoisomerase IV, which are type II topoisomerases essential for DNA replication, transcription, repair, and recombination (Fish and Chow, 1997).

In humans, levofloxacin is not subject to extensive metabolism, as it is found unchanged in the urine. Indeed, approximately 79.6% of the administered dose is recovered as original drug within 24 h of administration. Three metabolites have been identified at low concentrations, levofloxacin-β-D-glucuronide (M1), desmethyl-levofloxacin (M2), and levofloxacin-N-oxide (M3), although only M2 and M3 were detected in humans (Fish and Chow, 1997; Levaquin, 2008).

At therapeutic plasma concentrations levofloxacin does not alter CYP450 enzymes activity, thereby not being relevant for drug interactions at this level (Levaquin, 2008; Fàbrega et al., 2009).

Moxifloxacin is a fluoroquinolone antibiotic with high potency and superior penetration into tissues and lesions compared to levofloxacin, making it effective for TB treatment. By inhibiting DNA gyrase and topoisomerase IV, moxifloxacin hinders DNA replication and transcription. Its efficacy has been confirmed in a phase 3 clinical trial for RR-TB, thus representing a key component of the shortened treatment regimen for DS-TB (Sarathy et al., 2019; Nunn et al., 2019; Ahmad et al., 2018).

Approximately 52% of an oral or intravenous dose is metabolized via glucuronide and sulfate conjugation. Moxifloxacin is not substrate of the CYP450 system, nor does it affect such enzymes, minimizing its interaction with other drugs. The main metabolites are the sulfate conjugate (M1), eliminated in faeces, and the glucuronide conjugate (M2), excreted in the urine. About 45% of the dose is excreted as unchanged drug (Avelox, 2016; Strydom et al., 2019; Pienaar et al., 2017).

Linezolid is the first member of the oxazolidinone antibiotic class and acts by inhibiting bacterial protein synthesis. It binds to the 50S ribosomal subunit, preventing the formation of the functional 70S initiation complex required for translation (Leach et al., 2011; Shinabarger et al., 1997).

Linezolid undergoes metabolism primarily through non-enzymatic oxidation of its morpholine ring, resulting in two inactive metabolites: aminoethoxyacetic acid (Metabolite A), the predominant one, and hydroxyethyl glycine (Metabolite B). Importantly, the process does not involve the CYP450 enzyme system, showing no inhibitory or inductive effect on clinically significant isoforms such as CYP1A2, CYP2C9, CYP2C19, CYP2D6, CYP2E1, or CYP3A4. The absence of interaction with these major isoforms significantly reduces the risk of CYP450-mediated DDIs (Dryden, 2011; Zyvox, 2014; Slatter et al., 2001). Conversely, a potential for interactions with drugs affecting monoamine levels exists, as it is a reversible, non-selective inhibitor of monoamine oxidase (MAO) (Zyvox, 2014).

Excretion is primarily renal, with approximately 84% of an administered dose excreted in the urine (30% as the unchanged drug, 40% as Metabolite B, and 10% as Metabolite A). Faecal elimination is minimal, accounting for only small amounts of metabolites (Zyvox, 2014; Slatter et al., 2001).

Ethionamide is commonly used in longer regimens, especially when other drugs like bedaquiline, clofazimine, delamanid, or linezolid are not feasible. Its bacteriostatic or bactericidal effects depend on drug concentration at the infection site and organism susceptibility. Ethionamide requires activation by the mycobacterial enzyme flavin monooxygenase (EthA) and the transcriptional repressor EthR to form ethionamide sulfoxide, which binds to NAD^+^ and inhibits the enoyl-acyl carrier protein reductase (InhA), thereby blocking mycolic acid synthesis, responsible for cell death (Dover et al., 2007).

Ethionamide is extensively metabolized in the liver, mainly through flavin-containing monooxygenase (FMO3). Six metabolites have been identified: 2-ethylisonicotinamide, carbonyl-dihydropyridine, thiocarbonyl-dihydropyridine, S-oxocarbamoyl dihydropyridine, 2-ethylthioisonicotinamide, and ethionamide sulfoxide, the latter having significant antimycobacterial activity (Henderson et al., 2008; Krueger and Williams, 2005; Trecator, 2016).

Clofazimine, originally an anti-leprosy antibiotic, is now recommended as a core drug in both short and long regimens for DR-TB. Although the exact mechanism of action is not fully understood, clofazimine seems to interfere with cellular membrane functions, including ion transport and respiration, ultimately causing Mtb death (Cholo et al., 2017; Barry et al., 1956; Yano et al., 2011; Lechartier and Cole, 2015).

Clofazimine metabolism comprises partial hepatic transformation, with at least eight metabolites identified in human liver microsomes. CYP3A4 and CYP1A2 are primarily involved, with additional contributions from CYP2C8 and CYP2D6. At low concentrations, it is a weak inducer of CYP3A4, but at therapeutic levels shows inhibitory properties, suggesting the potential for both auto-induction and inhibition depending on the concentration (Howlader et al., 2022; Shimokawa et al., 2015). Notably, *in vitro* studies report that clofazimine also inhibits CYP2C8, CYP2D6, and CYP3A4/5, indicating the likelihood of clinically significant interactions with drugs metabolised by these isoenzymes (Lamprene, 2019; Maartens et al., 2018).

Renal excretion is minimal, with most of clofazimine and its metabolites eliminated through biliary system. The main urinary metabolites, apparently pharmacologically inactive, are produced by hydrolytic dehalogenation and deamination followed by glucuronidation (Banerjee et al., 1974; Levy, 1974; P. C. Feng, Fenselau, and Jacobson, 1981).

Bedaquiline, a diarylquinoline antimycobacterial drug, targets Mtb via inhibition of ATP synthase, essential for bacterial survival. Specifically, it binds to enzyme subunit c. Impeding this crucial mechanism of energy production, bedaquiline is particularly effective against persistent bacilli (Sirturo, 2012; Mesens et al., 2010).

It is predominantly metabolised by CYP3A4 in the liver into the N-monodesmethyl (M2) metabolite, which exhibits anti-tubercular activity, albeit with approximately five times less potency than the parent drug. Minor contributions to drug metabolism have been observed *in vitro* from CYP1A1, CYP2C8, and CYP2C18, though these enzymes are less relevant *in vivo* due to their lower hepatic expression compared to CYP3A4 (Shimada et al., 1994). Bedaquiline does not induce or inhibit major CYP isoenzymes, namely, CYP1A2, CYP2C9, CYP2C19, or CYP2D6 at clinically relevant concentrations, minimizing the risk of CYP450-related interactions (Mesens et al., 2010). Moreover, its impact on isoforms CYP1A2, CYP2A6, CYP2C8/9/10, CYP2C19, CYP2D6, CYP2E1, CYP3A4, CYP3A4/5, and CYP4A has also been shown to be negligible *in vitro.*

Excretion is primarily biliary, with a minimal amount appearing in the urine. The drug does not significantly interact with transport proteins like P-gp, further reducing the risk of DDIs (Sirturo, 2012).

Pretomanid is a prodrug used in combination with bedaquiline and linezolid for tackling pulmonary XDR-TB and treatment-intolerant or nonresponsive MDR-TB. It requires activation by Mtb deazaflavin-dependent nitroreductase (Ddn). Under aerobic conditions, pretomanid stops protein and lipid synthesis by reducing the availability of keto mycolic acids, essential for cell wall integrity. In anaerobic environments, it produces nitric oxide (NO), which inhibits cytochrome c oxidase and reduces ATP production in non-replicating Mtb cells (Pstragowski, Zbrzezna, and Bujalska-Zadrozny, 2017; Singh et al., 2008; Malo et al., 2021).

Approximately 20% of drug metabolism is due to partial phase one reactions by CYP3A4. At clinical concentrations, pretomanid does not inhibit major CYP isoenzymes, including CYP1A2, CYP2C8, CYP2C9, CYP2C19 or CYP2D6, and does not induce CYP2C9 or CYP3A4, limiting the potential for DDI trough CYPs. On the other hand, it significantly inhibits the OAT3 transporter, potentially increasing the concentration of OAT3 substrates (Mitnick, McGee, and Peloquin, 2009; Pretomanid, 2019). Of an administered dose, about 53% is excreted in the urine and 38% in faeces, both as unchanged drug and metabolites (Wang et al., 2015).

Delamanid is a prodrug indicated as part of a combination regimen for treating MDR-TB in adults. It requires activation by the mycobacterial F420 coenzyme system, including Ddn, to exert its antimycobacterial activity. Upon activation, it inhibits the synthesis of methoxy-mycolic and keto-mycolic acids, leading to depletion of essential cell wall components and subsequent bacterial death (Lewis and Sloan, 2015; Szumowski and Lynch, 2015).

Delamanid is primarily metabolized by albumin and, to a lesser extent, by CYP3A4 in the liver. Secondary metabolic pathways may involve CYP1A1, CYP2D6, and CYP2E1, though their contributions are minor in comparison to CYP3A4. Its main metabolite, DM-6705 (M1), accounts for 13%–18% of total plasma exposure and has been associated with QT prolongation. The hydrolytic cleavage of its 6-nitro-2,3-dihydroimidazo [2,1-b] oxazole moiety generates M1, which undergoes further transformations involving hydroxylation and oxidation, mediated largely by CYP3A4 (Szumowski and Lynch, 2015; Deltyba, 2014). While M1 and other metabolites do not retain significant antimycobacterial activity, they can still impact cardiac safety. Delamanid is primarily cleared via hepatic metabolism, with negligible renal excretion (Deltyba, 2014; Sasahara et al., 2015).

Among antituberculous medications, INH, RIF, EMB, clofazimine, bedaquiline, and delamanid are known to be CYP450 enzyme system substrates or can impact its metabolic activity by induction or inhibition. Therefore, potential interactions with cigarette smoking compounds may be expected.

Given the activity of PAHs in inducing CYP2E1, smokers taking INH as part of TB therapy may experience increased synthesis of reactive intermediates resulting from further oxidation of oxidised HZ and AcHZ. This could increase the risk of INH-associated hepatotoxicity. However, most studies do not include cigarette smoking as a possible individual risk factor for drug-induced hepatotoxicity (DIH). Interestingly, a retrospective cohort study highlighted that cigarette smoking appears to be negatively associated with DIH, which is surprising as induction of CYP2E1 in smokers is expected to increase exposure to toxic metabolites (Zaverucha-do-Valle et al., 2014). The reduced risk may be related to tobacco smoke compounds in glutathione S-transferase (GST) activity, which is involved in the nicotine detoxification pathway (Pachauri and Flora, 2013). Another explanation for this phenomenon could be a link between smoking habit and NAT2 acetylator status. Indeed, heterocyclic amines in tobacco smoke require activation by CYP1A2 and NAT2 (Voutsinas et al., 2013). Slow acetylator status is associated with reduced ability to detoxify these xenobiotics, but active smokers may have higher NAT2 activity and faster INH metabolism (Kroon, 2007). Based on these premises, smokers who quit may be at increased risk of developing DIH and thus may require dose adjustment to avoid liver damage.

RIF induction of CYP3A4 is mediated by an orphan nuclear receptor known as the pregnane X receptor (PXR). The drug binds to PXR, forming an activated complex, and subsequently combines with the retinoid X receptor (RXR) to form a heterodimer that targets a DNA response element, enhancing CYP3A4 gene transcription. Consequently, CYP3A4 protein synthesis is increased (Lehmann et al., 1998). The expression of several other proteins is induced in a similar manner. Indeed, genes reported to be regulated by known PXR ligands are CYP1A1, CYP2C8, CYP2C9, MDR1, MRP2, and members of the UGT, sulfotransferase, and carboxylesterase families (Goodwin, Redinbo, and Kliewer, 2002). Moreover, *in vitro* studies have demonstrated that RIF induces various CYP enzymes, including CYP1A2, CYP2A6, CYP2B6, CYP2C8, CYP2C9, CYP2C19, and CYP3A5 (Rae et al., 2001; Dalet-Beluche et al., 1992; Niemi et al., 2003). Concerning CYP1A2, *in vivo* induction is suggested, as demonstrated by the paraxanthine-to-caffeine ratio rising in healthy subjects (Branch et al., 2000; Fuhr and Rost, 1994).

In light of these findings, the safety and efficacy profile of RIF during TB treatment can be altered in smokers because of the concomitant induction of CYP450 enzymes mediated by PAHs. It is worth mention that RIF clearance increases during multiple-dose therapy, leading to autoinduction of its own metabolism, further complicating therapeutic management (Loos et al., 1987).

According to Lee S.Y. and colleagues, EMB has strong inhibitory potential against CYP1A2 and CYP2E1. Conversely, PAHs are inducers of such cytochromes. Therefore, careful assessment of safety and efficacy profiles of co-administered medications in TB patients who also smoke are warranted to avoid ADRs and achieve therapeutic goals.

Clofazimine is at least partially metabolised in the liver via CYP3A4 and CYP1A2, thereby TB smokers may experience increased metabolite synthesis, potentially necessitating dosage adjustments.

As bedaquiline mainly undergoes phase one reactions catalysed by CYP3A4, its exposure may be either reduced or increased during co-administration with inducers or inhibitors of CYP3A4 respectively (Sirturo, 2012). To a lesser extent, CYP1A1 also participates in drug metabolism, thus co-administering CYP1A1 inducers can decrease bedaquiline concentrations neglecting its effectiveness.

The metabolic pathways of delamanid involve CYP450 isoenzymes as well. In particular, CYP1A1 and 2E1, although playing a minor role in the overall metabolism, can be induced by PAHs making smokers more prone to severe ADRs (e.g., QT prolongation) due to increased metabolite concentrations.

**FIGURE 1:**
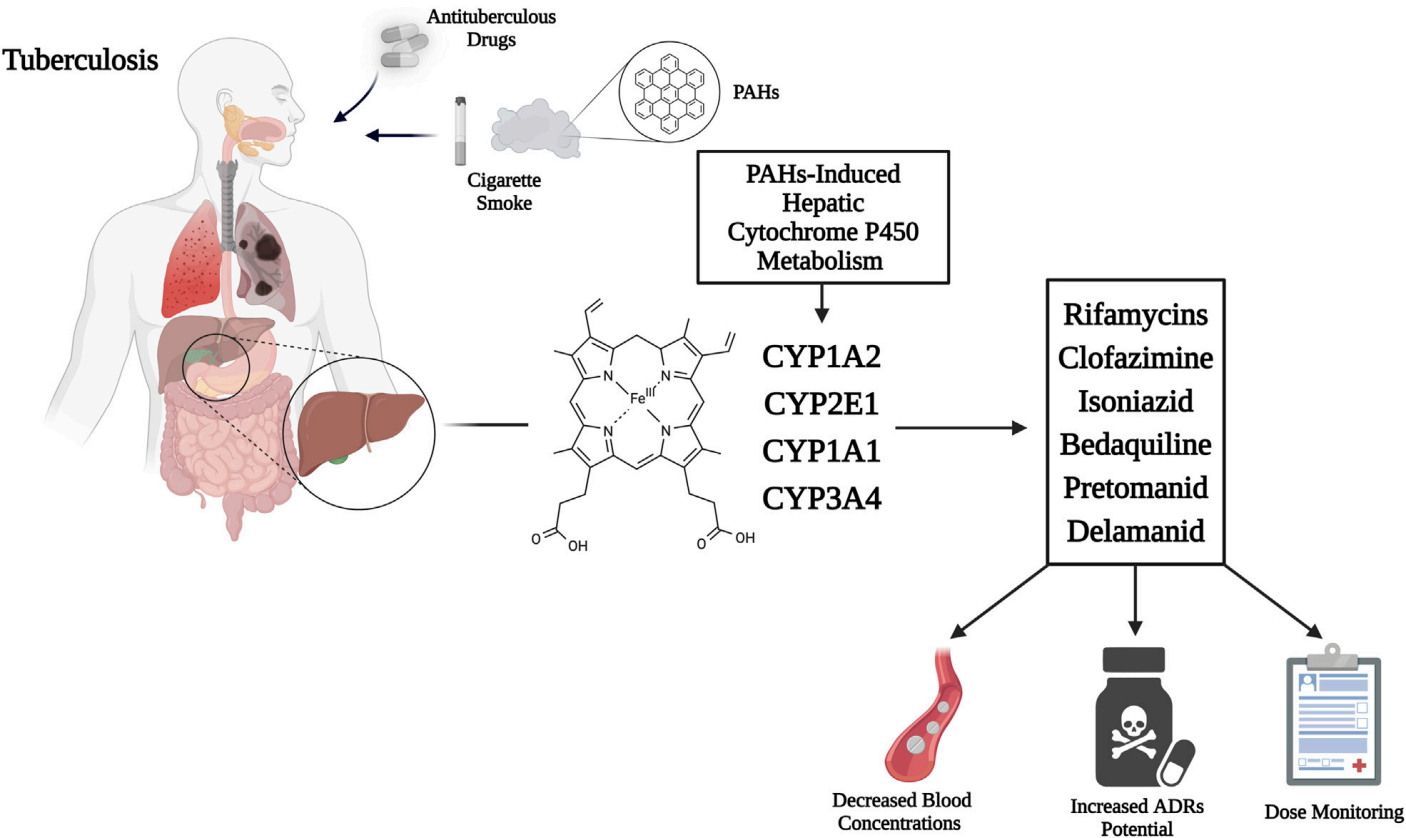
Mechanism of CYP450 hepatic isoenzymes induction by cigarette smoke PAHs, enhancing rifamycins, clofazimine, isoniazid, bedaquiline, pretomanid, and delamanid metabolism, thus decreasing active drug concentrations, increasing toxic metabolites and potential for ADRs. Therefore, dose monitoring appears necessary. Abbreviations: CYP450, cytochrome P450; PAHs, polycyclic aromatic hydrocarbons; ADRs, adverse drug reactions. Created with BioRender.com; accessed on 20 June 2025.

Table 1 and Figure 1 provide an overview of the antituberculous medications known to be CYP450 enzyme system substrates or can impact its metabolic activity and possible interactions with tobacco smoke compounds.

**TABLE 1:** Antituberculous medications known to be CYP450 enzyme system substrates or can impact its metabolic activity, possible effects of tobacco smoke compounds, and resulting potential interactions.

Drug	Hepatic CYP450 Metabolic Pathway Involved	Tobacco Smoke Compounds Effects on CYP450 Activities (Ref.)	Resulting Potential Interactions
Isoniazid	CYP2E1 oxidates its metabolites	PAHs may contribute to CYP2E1 induction	Given the activity of PAHs in inducing CYP2E1, smokers taking INH as part of TB therapy may experience increased synthesis of reactive intermediates resulting from further oxidation of oxidised HZ and AcHZ. This could increase the risk of INH-associated hepatotoxicity. However, most studies do not include cigarette smoking as a possible individual risk factor for drug-induced hepatotoxicity (DIH)
Rifampin	The drug itself is an inducer of CYP3A4, CYP1A2, CYP2B6, CYP2C8, CYP2C9, CYP2C19	Concomitant induction of CYP450 enzymes mediated by PAHs	Safety and efficacy profile of rifampin during TB treatment can be altered in smokers because of the concomitant induction of CYP450 enzymes mediated by PAHs. It is worth mention that rifampin clearance increases during multiple-dose therapy, leading to autoinduction of its own metabolism, further complicating therapeutic management
Ethambutol	The drug itself is a strong inhibitor of CYP1A2 and CYP2E1	PAHs are inducers of CYP1A2 and CYP2E1	Careful assessment of safety and efficacy profiles of co-administered medications in TB patients who also smoke are warranted to avoid ADRs and achieve therapeutic goals
Rifabutin	The drug itself is a mild inducer of CYP450 enzymes	Concomitant induction of CYP450 enzymes mediated by PAHs	Safety and efficacy profile of rifabutin during TB treatment can be altered in smokers because of the concomitant induction of CYP450 enzymes mediated by PAHs
Rifapentine	The drug itself is an inducer of CYP3A4 and CYP2C8/9	*In vitro* study highlighted that PAHs activate CYP3A4 gene transcription through the activation of hPXR in HepG2 cells. Thus, PAHs may contribute to CYP3A4 induction in human liver (Kumagai et al., 2012)	Safety and efficacy profile of rifapentine during TB treatment can be altered in smokers because of the concomitant induction of CYP450 enzymes mediated by PAHs
Clofazimine	It is primarily metabolised by CYP3A4 and CYP1A2, with additional contributions from CYP2C8 and CYP2D6. Moreover, at low concentrations, the drug itself is a weak inducer of CYP3A4, but at therapeutic levels shows inhibitory properties, suggesting the potential for both auto-induction and inhibition depending on drug concentration	PAHs are inducers of CYP1A2 and may also contribute to CYP3A4 induction in human liver (Kumagai et al., 2012)	TB smokers may experience increased metabolite synthesis, potentially necessitating dosage adjustments. Furthermore, adequate dose monitoring is even more important when considering that clofazimine is itself, at low concentration, a weak inducer of CYP3A4, but at therapeutic levels shows inhibitory properties
Bedaquiline	Metabolised by CYP3A4 and CYP1A1	PAHs are inducers of CYP1A1 and may also contribute to CYP3A4 induction in human liver (Kumagai et al., 2012)	Bedaquiline exposure may be reduced during co-administration with CYP1A1 and CYP3A4 inducers like PAHs, neglecting its effectiveness
Pretomanid	Partially metabolised by CYP3A4	PAHs may contribute to CYP3A4 induction in human liver (Kumagai et al., 2012)	Although pretomanid is just partially metabolised by CYP3A4, its exposure may be reduced during co-administration with CYP3A4 inducers like PAHs, neglecting its effectiveness
Delamanid	Metabolised by CYP3A4 and, to a lesser extent, by CYP1A1, CYP2D6, and CYP2E1	PAHs are inducers of CYP1A1 and CYP2E1. Moreover, they may contribute to CYP3A4 induction in human liver (Kumagai et al., 2012)	CYP1A1 and 2E1, although playing a minor role in the overall delamanid metabolism, can be induced by PAHs making smokers more prone to severe ADRs (e.g., QT prolongation) due to increased metabolite concentrations

## 5 Interactions between smoking cessation medications and antituberculous drugs

In the past few years, numerous studies have investigated the association between smoking and TB severity suggesting that the former has a negative impact on patients’ outcome, resulting in delayed culture conversion, treatment extension, and increasing risk of recurrence after pharmacotherapies completion (Slama et al., 2007; Smit et al., 2010; Abal et al., 2005; Nijenbandring de Boer et al., 2014). In this context, Altet N. et al. evaluated the effect of tobacco smoke on radiological manifestations, sputum conversion, and immune response to Mtb by analysing IFN-γ secretion using IFN-γ Release Assays (IGRAs). To this aim, 525 participants were studied: 175 with active pulmonary TB, 350 from contact tracing studies, and 41 with secondary TB cases. Clinical, radiological, and microbiological data were collected for each participant, and they underwent QuantiFERON-TB Gold (QFN-G-IT) and T-SPOT.TB. The authors inferred that smoking had a negative effect on radiological manifestations and delayed the time to sputum conversion, probably related to an attenuated IFN-γ response caused by direct tobacco smoke (Altet et al., 2017).

A prospective cohort study has been performed in India to determine the impact of smoking on TB treatment outcome and the prevalence of smoking habits among newly diagnosed individuals. The investigators enrolled 2,350 patients (1,758 male and 592 female) who were classified as never smokers, current smokers, and former smokers on the basis of self-report. Participants were then started on anti-TB treatment and followed for 2 years. During the observation period, smoking has been associated with more extensive lung disease, cavitation, and positive sputum smear and culture results at baseline. Additionally, both current and former smokers were significantly more likely to have positive microbiological tests after 2 months of treatment. The same groups also showed higher rates of non-adherence, treatment failure, and relapse. In light of these findings, it has been concluded that tobacco smoking is associated with a significantly increased risk of advanced and more severe disease in the form of pulmonary cavitation, positive sputum smear and culture results, and lower conversion rate after treatment initiation (Mahishale et al., 2015).

In 2022, a prospective observational study came to opposite conclusions though. The purpose of the study was to describe the severity of disease and treatment outcomes in TB smokers compared with non-smokers in Guinea-Bissau. It showed that among the 1,780 patients included, 385 of whom were smokers for a median of 10 years, there was no difference in disease severity at diagnosis. In addition, smokers were not more prone to negative treatment outcomes, although a trend was observed (adjusted odds ratio [OR] 1.24, 95% confidence interval [CI] 0.91–1.70), and were more likely to be lost to follow-up, but this was also not significant (adjusted hazard ratio [HR] 2.09, 95% CI 0.89–4.94). Thus, the authors suggested that in a highly endemic TB setting with few tobacco smokers, worse disease severity or outcome due to smoking were not observed, but they highlighted the existence of confounding factors (Bay et al., 2022).

Another study, conducted in Taipei, sought to determine whether tobacco smoking increases the risk of TB relapse in adults who have successfully completed TB treatment, and the underlying factors. A total of 5,567 patients were enrolled, of whom 84 (1.5%) experienced recurrence during follow-up. The incidence of relapses was 4.9 episodes/1,000 person-years of follow-up. In subjects who smoked more than ten cigarettes per day, the risk was twice that of non-smokers/former smokers, according to Cox proportional hazards regression (Yen et al., 2014).

Despite mounting evidence of an association between smoking and TB in terms of risk and influence on outcome, a crucial but often overlooked issue is the interaction between antituberculosis drugs and smoking cessation therapies. Indeed, a significant number of people affected by TB are also smokers in the process of quitting. Unveiling the molecular mechanisms that underscore these events is of utmost importance, as clinically relevant DDIs can complicate TB management and interfere with the success of smoking cessation.

Interactions can also have a significant impact on the safety and efficacy profile of treatments, particularly if they compromise pharmacological activity and increase the odds of ADRs.

Medications for smoking cessation include various forms of nicotine replacement therapy (NRT), bupropion, varenicline, and cytisine (Polosa and Benowitz, 2011; Cohen et al., 2024) that have been shown to improve quit rates in the general population of smokers. Compared with placebo, the likelihood of quitting smoking was roughly doubled with NRT (1.84, 95% CI 1.71–1.99) and bupropion (1.82, 1.60–2.06) and was improved further with varenicline (2.88, 2.40–3.47) (Cahill et al., 2013).

Dogar O. et al. conducted a randomised, double-blind, placebo-controlled trial to assess the effectiveness and safety of cytisine in patients with TB in Bangladesh and Pakistan. 2,472 patients (1,527 from Bangladesh and 945 from Pakistan) smoking daily and willing to quit who had been diagnosed with pulmonary TB within the previous 4 weeks, were enrolled. Subjects were randomly assigned to receive cytisine (n = 1239) at a dose of 9 mg on day 0, gradually tapered to 1.5 mg on day 25, or placebo (n = 1233) for 25 days. The primary endpoint was continuous abstinence at 6 months, defined as self-report and confirmed biochemically by a breath CO reading of less than 10 parts per million (ppm). Primary and safety analysis were done in the intention-to-treat (ITT) population. At 6 months, 401 (32.4%) participants in the cytisine group and 366 (29.7%) in the placebo group had achieved continuous abstinence. Fifty-three (4.3%) patients in the cytisine group and 46 (3.7%) in the placebo group reported serious adverse events (SAEs) (94 events in the cytisine group and 90 events in the placebo group), which included 91 deaths (49 in the cytisine group and 42 in the placebo group). None of the adverse events (AEs) were attributed to the study medication. Authors stated that the add-on of cytisine to brief behavioural support for the treatment of tobacco addiction in TB patients is not supported by their findings (Dogar et al., 2020).

An open-label randomised controlled trial investigated the impact of intensive smoking cessation activities as an add-on to TB treatments on patient-related outcomes. A total of 800 self-reporting smokers with pulmonary TB on standard anti-TB drugs were enrolled in the study and randomly assigned (1:1) to receive either NRT plus behavioural change counselling or counselling alone, delivered at baseline and at two follow-up visits. The primary endpoints were change in TB-score at 24 weeks and culture conversion at 8 weeks. The biochemical smoking cessation rates, defined as serum cotinine levels of less than 10 ng/mL and/or exhaled CO levels of less than 6 ppm, were significantly higher in the intervention group at 24 weeks (47.8% vs. 32.4%) as well as the self-reported cessation rates (69.3% vs. 38.7%). Despite the TB-scores at 24 weeks (95% CI) were lower in the intervention arm (2.07 vs. 2.12), the difference was not clinically meaningful. Patients in the control arm required treatment extension more often than intervention arm (6.4% vs. 2.6%). Authors concluded that combining NRT with behaviour change counselling resulted in a significantly higher quit rates and lower cotinine levels, but significant impact on patient-related or microbiological outcomes were not detected (Sharma et al., 2018).

Safety concerns arise when smoking cessation medications and anti-TB drugs are co-administered, so predicting and assessing potential DDIs would be extremely helpful. To date, many digital tools are available to determine their clinical significance, being the management largely dependent on impact and severity of the interaction. However, there is no consensus among the current resources and a standardised classification method would be warranted. More specifically, the British National Formulary marks with bullet points potentially harmful drug pairs which should be prescribed cautiously, under appropriate monitoring, or avoided altogether (British National Formulary, 2015). Micromedex Drug–Reax System categorizes interactions into three degrees of severity, major, moderate, and minor, and the strength of the reporting into five categories—excellent, good, fair, poor, and unlikely (DRUG-REAX, 2024). Drugs.com Drug Interaction Checker (DDIC) and DrugBank.com classify interactions into four severity levels: major, moderate, minor, and unknown (Knox et al., 2024; Drug Interaction Checker Quickly Check Your Meds, 2024). Vidal’s Interactions médicamenteuses comprises four seriousness grades according to the recommended clinical management—contraindicated, avoid, precaution, and “take into account” (i.e., no specific recommendation) (Les interactions médicamenteuses, 2024). Drug Interaction Facts rates interaction severity into three levels—major, moderate, and minor—and the degree of documentation into five—established, probable, suspected, possible, and unlikely—by combining these two categories. It also ranks each interaction from 1 to 5 in terms of importance (Tatro, 2014). MedScape drug interaction checker sorts DDIs as serious, significant, or minor. Serious interactions impose switching to an alternative molecule, whereas significant ones necessitate close monitoring. Minor DDIs do not require either discontinuation or switching (Drug Interactions Checker - Medscape Drug Reference Database, 2024). Notably, information from digital tools must always be compared with SmPC of interacting drugs in order to avoid errors or misinterpretations.

Concerning potential DDIs between NRT and anti-TB drugs, DrugBank.com reports that the metabolism of nicotine can be decreased when combined with INH or EMB and increased when co-administered with rifabutin. Indeed, when nicotine is administered concurrently with a CYP2A6 inhibitor of unknown strength, the substrate metabolism will be reduced, resulting in increasing serum concentrations and heightened risk, incidence, and/or severity of ADRs. Conversely, concomitant use of nicotine and rifabutin, a CYP2B6 inducer, will result in a modest increase in mediated metabolism of the substrate, potentially leading to a reduction in serum concentration and/or therapeutic effect (DrugBank Clinical API Plugins, 2024).

*In vitro* research suggests that varenicline does not appear to influence the pharmacokinetics of substances that are primarily metabolised by CYP enzymes. Since less than 10% of its clearance is attributed to varenicline’s metabolism, it is anticipated that agents affecting the CYP450 system will have no effect on its pharmacokinetic profile. This suggests that dose adjustments will not be necessary. Furthermore, at therapeutic levels, varenicline does not inhibit human renal transport proteins, as demonstrated by preclinical studies. Consequently, it is improbable that active substances eliminated by renal secretion will be impacted by varenicline (Champix, 2006). As indicated by online sources, INH, PZA, EMB, and levofloxacin have the potential to reduce its excretion rate. Conversely, varenicline may also impact the clearance of streptomycin, capreomycin, and amikacin. The renal clearance of medications is influenced by various renal functions, including glomerular filtration, passive diffusion, tubular secretion, and reabsorption. It is important to note that two of these processes, namely, tubular secretion and reabsorption, are subject to saturation. As a result, they can be influenced by competition among several substrates for removal. This suggests a significant likelihood that one drug will “out-compete” or saturate the renal excretion pathways prior to the elimination of the other co-administered drugs, leading to inhibited or delayed clearance. The net result of these processes is an increase in serum concentrations, which, by the way, has been demonstrated to escalate the risk and/or severity of ADRs associated to exposure to such drugs (DrugBank Clinical API Plugins, 2024).

Bupropion is subject to metabolic processes involving the CYP450 enzyme system, predominantly through CYP2B6, with contributions from CYP1A2, 2A6, 2C9, 2D6, 2E1, and 3A4 isoforms, though to a lesser degree (Connarn et al., 2015). These enzymes also play a pivotal role in the metabolic pathways of various TB medications, which could lead to alterations in the concentrations and effects of such drugs. It is imperative to adjust dosages and meticulously select treatments for patients who are using bupropion. Furthermore, research from both preclinical and clinical studies indicates that bupropion can inhibit CYP2D6 activity. The oxidation of the bupropion side chain leads to the production of a glycine conjugate of metachlorobenzoic acid, which is primarily expelled as the main urinary metabolite, with glucuronidated metabolites also being found in urine. Although there are discrepancies in the literature regarding their various chiral forms, these metabolites are produced from all three active metabolites by different UGT enzymes. *In vitro*, the primary enzyme involved in the glucuronidation of hydroxybupropion is UGT2B7, with a minor role played by UGT2B4. UGT2B7 is also chiefly responsible for generating erythrohydrobupropion glucuronide, while UGT1A4, UGT1A3, UGT1A9, and UGT2B4 have lesser involvement. The identification of the UGTs that mediate the conjugation of active bupropion metabolites is crucial for understanding factors that could affect potential DDIs. *In vitro* research has demonstrated that at concentrations up to 200 mcg/ml, 84% of bupropion binds to proteins in human plasma. Hydroxybupropion exhibits a similar binding rate, whereas the protein binding extent for threohydrobupropion is about half that of bupropion. Consequently, interactions related to protein binding are unlikely to have clinical significance (Jefferson, Pradko, and Muir, 2005).

Digital tools highlight that potential DDIs between bupropion and anti-TB drugs, namely, RIF, INH, PZA, EMB, rifabutin, streptomycin, capreomycin, amikacin, levofloxacin, moxifloxacin, linezolid, and bedaquiline may occur.

DDIC, Drugs.com, MedScape.com, and INTERCheckWeb.it points out the plausible interaction between bupropion and RIF. In particular, according to DDIC and MedScape.com, RIF will decrease the level or effect of bupropion by inducing CYP2B6 activity. Therefore, pharmacological response to bupropion should be monitored more closely whenever a CYP2B6 inducer is added to or withdrawn from therapy, and the bupropion dosage adjusted as necessary. DrugBank.com reports that the metabolism of bupropion can be increased when combined with RIF. Indeed, the latter is an inducer of CYP2C9 while bupropion is substrate of CYP2C9. Concomitant administration of these agents can produce a decrease in the serum concentration of the affected drug which may translate to reduced therapeutic efficacy. Ultimately, INTERCheckWeb.it suggests a reduction in systemic exposure to bupropion with an associated increase in clearance of 203% and reductions in Cmax and AUC of 39% and 43% respectively, due to the rifampin-related induction of bupropion metabolism.

Two digital tools identified the potential interaction between bupropion and INH. DrugBank.com reports that the risk or severity of seizure can be increased when bupropion is combined with INH. Indeed, bupropion carries a dose-dependent risk of seizure further exacerbated when combined with other medications that can reduce the seizure threshold. According to Drug.com, combining INH with other medications known to cause liver toxicity, such as bupropion, could increase the likelihood of liver injury. INH is subject to metabolism through the actions of N-acetyltransferase and CYP2E1. The acetylation process of INH varies genetically and is generally categorized as either slow or rapid, with slow acetylators showing a relative deficiency in N-acetyltransferase. While the rate of acetylation does not significantly impact the drug’s effectiveness, it may result in elevated levels of INH in the bloodstream and a higher occurrence of ADRs. Furthermore, INH has been found to act as an *in vitro* inhibitor for several isoenzymes, including CYP450 (2C9, 2C19, 2E1, and 3A4). Consequently, concurrent administration of hepatotoxic medications that are metabolised via these pathways may result in elevated concentrations of the concomitant drug, which can potentially culminate in hepatic injury.

PZA, EMB and levofloxacin have been demonstrated to reduce the renal clearance rate of bupropion, with the potential observation of elevated plasma levels. Consequently, this may result in an escalation of the risk, occurrence, and/or severity of ADRs associated with bupropion exposure. Conversely, bupropion has been observed to inhibit the renal elimination of streptomycin, capreomycin, and amikacin, which could result in elevated serum concentrations and an augmented risk of ADRs associated with aminoglycosides (DrugBank Clinical API Plugins, 2024).

Regarding the co-administration of bupropion with linezolid, four tools indicate an increased risk of serotonergic syndrome (agitation, mental confusion, cognitive deficits, diaphoresis, myoclonus, hyperreflexia, hypertension, palpitations, and muscle rigidity). Therefore, it would be recommended to use the combination with caution and to monitor the onset of early signs such as anxiety, confusion, disorientation and, if necessary, to discontinue drug treatment (Drug Interaction Checker Quickly Check Your Meds, 2024; Drug Interactions Checker - Medscape Drug Reference Database, 2024; DrugBank Clinical API Plugins, 2024; Intercheck WEB, 2024).

Finally, co-administration of bedaquiline with bupropion may cause an increased risk of hepatotoxicity, so it is better to avoid this association whenever possible, especially in patients who already have impaired liver function (Drug Interaction Checker Quickly Check Your Meds, 2024).

Possible interactions between antituberculous drugs and smoking cessation medications are summarised in Table 2.

**TABLE 2:** Possible interactions between antituberculous drugs and smoking cessation medications, classified according to severity level. Major/Serious: highly clinically significant. Avoid combinations; the risk of the interaction outweighs the benefit. Moderate: moderately clinically significant. Usually avoid combinations; use it only under special circumstances. Minor: minimally clinically significant. Minimize risk; assess risk and consider an alternative drug, take steps to circumvent the interaction risk and/or institute a monitoring plan. Unknown: no interaction information available.

Treatment Line	Drug	NRT (Ref.)	Bupropion (Ref.)	Varenicline (Ref.)	Cytisine (Ref.)
First Line	Rifampin	Unknown (Drug Interaction Checker Quickly Check Your Meds, 2024; Drug Interactions Checker - Medscape Drug Reference Database, 2024; DrugBank Clinical API Plugins, 2024; Intercheck WEB, 2024)	Moderate (Drug Interaction Checker Quickly Check Your Meds, 2024; Drug Interactions Checker - Medscape Drug Reference Database, 2024; DrugBank Clinical API Plugins, 2024; Intercheck WEB, 2024)	Unknown (Drug Interaction Checker Quickly Check Your Meds, 2024; Drug Interactions Checker - Medscape Drug Reference Database, 2024; DrugBank Clinical API Plugins, 2024; Intercheck WEB, 2024)	Unknown (Drug Interaction Checker Quickly Check Your Meds, 2024; Drug Interactions Checker - Medscape Drug Reference Database, 2024; DrugBank Clinical API Plugins, 2024; Intercheck WEB, 2024)
	Isoniazid	Moderate (DrugBank Clinical API Plugins, 2024)	Moderate (Drug Interaction Checker Quickly Check Your Meds, 2024; DrugBank Clinical API Plugins, 2024	Minor (DrugBank Clinical API Plugins, 2024)	Unknown (Drug Interaction Checker Quickly Check Your Meds, 2024; Drug Interactions Checker - Medscape Drug Reference Database, 2024; DrugBank Clinical API Plugins, 2024; Intercheck WEB, 2024)
	Pyrazinamide	Unknown (Drug Interaction Checker Quickly Check Your Meds, 2024; Drug Interactions Checker - Medscape Drug Reference Database, 2024; DrugBank Clinical API Plugins, 2024; Intercheck WEB, 2024)	Minor (DrugBank Clinical API Plugins, 2024)	Minor (DrugBank Clinical API Plugins, 2024)	Unknown (Drug Interaction Checker Quickly Check Your Meds, 2024; Drug Interactions Checker - Medscape Drug Reference Database, 2024; DrugBank Clinical API Plugins, 2024; Intercheck WEB, 2024)
	Ethambutol	Minor (DrugBank Clinical API Plugins, 2024)	Minor (DrugBank Clinical API Plugins, 2024)	Minor (DrugBank Clinical API Plugins, 2024)	Unknown (DrugBank Clinical API Plugins, 2024; Drug Interaction Checker Quickly Check Your Meds, 2024; Drug Interactions Checker - Medscape Drug Reference Database, 2024; Intercheck WEB, 2024)
	Rifabutin	Minor (DrugBank Clinical API Plugins, 2024)	Minor (DrugBank Clinical API Plugins, 2024)	Unknown (Drug Interaction Checker Quickly Check Your Meds, 2024; Drug Interactions Checker - Medscape Drug Reference Database, 2024; DrugBank Clinical API Plugins, 2024; Intercheck WEB, 2024)	Unknown (Drug Interaction Checker Quickly Check Your Meds, 2024; Drug Interactions Checker - Medscape Drug Reference Database, 2024; DrugBank Clinical API Plugins, 2024; Intercheck WEB, 2024)
	Rifapentine	Unknown (Drug Interaction Checker Quickly Check Your Meds, 2024; Drug Interactions Checker - Medscape Drug Reference Database, 2024; DrugBank Clinical API Plugins, 2024; Intercheck WEB, 2024)	Unknown (Drug Interaction Checker Quickly Check Your Meds, 2024; Drug Interactions Checker - Medscape Drug Reference Database, 2024; DrugBank Clinical API Plugins, 2024; Intercheck WEB, 2024)	Unknown (Drug Interaction Checker Quickly Check Your Meds, 2024; Drug Interactions Checker - Medscape Drug Reference Database, 2024; DrugBank Clinical API Plugins, 2024; Intercheck WEB, 2024)	Unknown (Drug Interaction Checker Quickly Check Your Meds, 2024; Drug Interactions Checker - Medscape Drug Reference Database, 2024; DrugBank Clinical API Plugins, 2024; Intercheck WEB, 2024)
Second Line	Streptomycin	Moderate (DrugBank Clinical API Plugins, 2024)	Moderate (DrugBank Clinical API Plugins, 2024)	Moderate (DrugBank Clinical API Plugins, 2024)	Unknown (Drug Interaction Checker Quickly Check Your Meds, 2024; Drug Interactions Checker - Medscape Drug Reference Database, 2024; DrugBank Clinical API Plugins, 2024; Intercheck WEB, 2024)
	Capreomycin	Moderate (DrugBank Clinical API Plugins, 2024)	Moderate (DrugBank Clinical API Plugins, 2024)	Moderate (DrugBank Clinical API Plugins, 2024)	Unknown (Drug Interaction Checker Quickly Check Your Meds, 2024; Drug Interactions Checker - Medscape Drug Reference Database, 2024; DrugBank Clinical API Plugins, 2024; Intercheck WEB, 2024)
	Amikacin	Minor (DrugBank Clinical API Plugins, 2024)	Moderate (DrugBank Clinical API Plugins, 2024)	Moderate (DrugBank Clinical API Plugins, 2024)	Unknown (Drug Interaction Checker Quickly Check Your Meds, 2024; Drug Interactions Checker - Medscape Drug Reference Database, 2024; DrugBank Clinical API Plugins, 2024; Intercheck WEB, 2024)
	Levofloxacin	Unknown (DrugBank Clinical API Plugins, 2024; Drug Interaction Checker Quickly Check Your Meds, 2024; Drug Interactions Checker - Medscape Drug Reference Database, 2024; Intercheck WEB, 2024)	Minor (DrugBank Clinical API Plugins, 2024)/Major (Drug Interaction Checker Quickly Check Your Meds, 2024)	Minor (DrugBank Clinical API Plugins, 2024)	Unknown (Drug Interaction Checker Quickly Check Your Meds, 2024; Drug Interactions Checker - Medscape Drug Reference Database, 2024; DrugBank Clinical API Plugins, 2024; Intercheck WEB, 2024)
	Moxifloxacin	Unknown (Drug Interaction Checker Quickly Check Your Meds, 2024; Drug Interactions Checker - Medscape Drug Reference Database, 2024; DrugBank Clinical API Plugins, 2024; Intercheck WEB, 2024)	Major (Drug Interaction Checker Quickly Check Your Meds, 2024)	Unknown (Drug Interaction Checker Quickly Check Your Meds, 2024; Drug Interactions Checker - Medscape Drug Reference Database, 2024; DrugBank Clinical API Plugins, 2024; Intercheck WEB, 2024)	Unknown (Drug Interaction Checker Quickly Check Your Meds, 2024; Drug Interactions Checker - Medscape Drug Reference Database, 2024; DrugBank Clinical API Plugins, 2024; Intercheck WEB, 2024)
	Linezolid	Unknown (Drug Interaction Checker Quickly Check Your Meds, 2024; Drug Interactions Checker - Medscape Drug Reference Database, 2024; DrugBank Clinical API Plugins, 2024; Intercheck WEB, 2024)	Serious (Drug Interactions Checker - Medscape Drug Reference Database, 2024)/Major (Drug Interaction Checker Quickly Check Your Meds, 2024; DrugBank Clinical API Plugins, 2024; Intercheck WEB, 2024)	Unknown (Drug Interaction Checker Quickly Check Your Meds, 2024; Drug Interactions Checker - Medscape Drug Reference Database, 2024; DrugBank Clinical API Plugins, 2024; Intercheck WEB, 2024)	Unknown (Drug Interaction Checker Quickly Check Your Meds, 2024; Drug Interactions Checker - Medscape Drug Reference Database, 2024; DrugBank Clinical API Plugins, 2024; Intercheck WEB, 2024)
	Ethionamide	Unknown (Drug Interaction Checker Quickly Check Your Meds, 2024; Drug Interactions Checker - Medscape Drug Reference Database, 2024; DrugBank Clinical API Plugins, 2024; Intercheck WEB, 2024)	Unknown (Drug Interaction Checker Quickly Check Your Meds, 2024; Drug Interactions Checker - Medscape Drug Reference Database, 2024; DrugBank Clinical API Plugins, 2024; Intercheck WEB, 2024)	Unknown (Drug Interaction Checker Quickly Check Your Meds, 2024; Drug Interactions Checker - Medscape Drug Reference Database, 2024; DrugBank Clinical API Plugins, 2024; Intercheck WEB, 2024)	Unknown (Drug Interaction Checker Quickly Check Your Meds, 2024; Drug Interactions Checker - Medscape Drug Reference Database, 2024; DrugBank Clinical API Plugins, 2024; Intercheck WEB, 2024)
	Clofazimine	Unknown (Drug Interaction Checker Quickly Check Your Meds, 2024; Drug Interactions Checker - Medscape Drug Reference Database, 2024; DrugBank Clinical API Plugins, 2024; Intercheck WEB, 2024)	Unknown (Drug Interaction Checker Quickly Check Your Meds, 2024; Drug Interactions Checker - Medscape Drug Reference Database, 2024; DrugBank Clinical API Plugins, 2024; Intercheck WEB, 2024)	Unknown (Drug Interaction Checker Quickly Check Your Meds, 2024; Drug Interactions Checker - Medscape Drug Reference Database, 2024; DrugBank Clinical API Plugins, 2024; Intercheck WEB, 2024)	Unknown (Drug Interaction Checker Quickly Check Your Meds, 2024; Drug Interactions Checker - Medscape Drug Reference Database, 2024; DrugBank Clinical API Plugins, 2024; Intercheck WEB, 2024)
	Bedaquiline	Unknown (Drug Interaction Checker Quickly Check Your Meds, 2024; Drug Interactions Checker - Medscape Drug Reference Database, 2024; DrugBank Clinical API Plugins, 2024; Intercheck WEB, 2024)	ModerateDrug Interaction Checker Quickly Check Your Meds, 2024)	Unknown (Drug Interaction Checker Quickly Check Your Meds, 2024; Drug Interactions Checker - Medscape Drug Reference Database, 2024; DrugBank Clinical API Plugins, 2024; Intercheck WEB, 2024)	Unknown (Drug Interaction Checker Quickly Check Your Meds, 2024; Drug Interactions Checker - Medscape Drug Reference Database, 2024; DrugBank Clinical API Plugins, 2024; Intercheck WEB, 2024)
	Pretomanid	Unknown (Drug Interaction Checker Quickly Check Your Meds, 2024; Drug Interactions Checker - Medscape Drug Reference Database, 2024; DrugBank Clinical API Plugins, 2024; Intercheck WEB, 2024)	Unknown (Drug Interaction Checker Quickly Check Your Meds, 2024; Drug Interactions Checker - Medscape Drug Reference Database, 2024; DrugBank Clinical API Plugins, 2024; Intercheck WEB, 2024)	Unknown (Drug Interaction Checker Quickly Check Your Meds, 2024; Drug Interactions Checker - Medscape Drug Reference Database, 2024; DrugBank Clinical API Plugins, 2024; Intercheck WEB, 2024)	Unknown (Drug Interaction Checker Quickly Check Your Meds, 2024; Drug Interactions Checker - Medscape Drug Reference Database, 2024; DrugBank Clinical API Plugins, 2024; Intercheck WEB, 2024)
	Delamanid	Unknown (Drug Interaction Checker Quickly Check Your Meds, 2024; Drug Interactions Checker - Medscape Drug Reference Database, 2024; DrugBank Clinical API Plugins, 2024; Intercheck WEB, 2024)	Unknown (Drug Interaction Checker Quickly Check Your Meds, 2024; Drug Interactions Checker - Medscape Drug Reference Database, 2024; DrugBank Clinical API Plugins, 2024; Intercheck WEB, 2024)	Unknown (Drug Interaction Checker Quickly Check Your Meds, 2024; Drug Interactions Checker - Medscape Drug Reference Database, 2024; DrugBank Clinical API Plugins, 2024; Intercheck WEB, 2024)	Unknown (Drug Interaction Checker Quickly Check Your Meds, 2024; Drug Interactions Checker - Medscape Drug Reference Database, 2024; DrugBank Clinical API Plugins, 2024; Intercheck WEB, 2024)

Table 3 summarise the role of non-CYP450 proteins involved in drug metabolism/transport and impact of cigarette smoking.

**TABLE 3:** Role of non-CYP450 proteins involved in drug metabolism/transport and impact of cigarette smoking on their activity.

Protein/Enzyme Family	Main Function	Relevant Drugs	Impact of Cigarette Smoking
Arylamine N-acetyltransferase 2 (NAT2)	Acetylation of drugs and xenobiotics	Isoniazid	Active smokers may have higher NAT2 activity, leading to faster INH metabolism
Uridine Diphosphate-glucuronosyltransferases (UGTs)	Phase II conjugation (glucuronidation) of drugs and their metabolites	Moxifloxacin, Levofloxacin (metabolites), Bupropion (metabolites)	The impact of PAHs from tobacco smoke is described as complex and unclear, with potential effects varying between different UGT isoforms
Flavin-containing monooxygenase 3 (FMO3)	Oxygenation and activation of certain drugs	Ethionamide	-
Amidase	Hydrolysis of amide bounds in prodrugs to their active forms	Isoniazid, Pyrazinamide	-
Xanthine Oxidase (XO)	Oxidation of PZA’s active metabolite (PA) to a hepatotoxic form (5-OH-PA)	Pyrazinamide	-
Aldehyde Dehydrogenase	Oxidation of aldehydes	Ethambutol	-
Transporter Proteins (e.g., OCTs, P-gp, MRP2, OAT3)	Mediate drug influx and efflux, affecting absorption, distribution, and renal/biliary excretion	Nicotine, Varenicline, Pretomanid (as OAT3 inhibitor), Rifampin (as P-gp/MRP2 inducer)	Nicotine itself interferes with Organic Cation Transporters (OCTs) *in vitro*. Competition for renal transporters can occur between drugs, potentially altering clearance

## 6 Discussion

To our knowledge this scoping review is the first to comprehensively explore the intricate relationship between tobacco smoking, smoking cessation, and TB treatment. Smoking plays a significant role in accelerating TB progression, worsening treatment outcomes, and increasing mortality rates. Furthermore, the impact of tobacco smoke on the enzymatic metabolism of antituberculous drugs highlights a critical challenge in optimizing treatment for TB patients who smoke. Smoking cessation, while beneficial for improving treatment outcomes, presents its own set of complexities, particularly in adjusting therapeutic regimens as enzymatic activity normalizes post-cessation. Careful monitoring of patients during this transition is therefore essential.

Tobacco smoke, particularly through its PAHs, alters the pharmacokinetics and pharmacodynamics of antituberculous drugs. By inducing hepatic enzyme activity, tobacco smoke accelerates drug metabolism, potentially reducing the efficacy of TB medications. Conversely, smoking cessation reverses this enzyme induction, necessitating tailored drug dosing to maintain therapeutic effectiveness while minimizing adverse reactions.

Evidence highlights the importance of integrated care strategies that combine TB treatment with smoking cessation efforts. Quitting smoking not only supports better TB outcomes but also reduces the risk of chronic conditions like cardiovascular disease and COPD. However, careful management of potential DDIs between anti-TB therapies and smoking cessation medications is essential to ensure both safety and efficacy. Monitoring interactions is critical when prescribing smoking cessation therapies alongside TB medications. Specifically, avoiding combinations such as bupropion with linezolid or bedaquiline is recommended due to the risk of serotonin syndrome or hepatotoxicity.

The present work has certain limitations, firstly because it is based on data from the existing literature, which appears to be fragmentary and inconsistent. Despite a careful search and selection of sources, the evidence often comes from works that were not designed to explore the field of drug-drug interactions and, in particular, with tobacco combustion compounds. Additionally, there is a paucity of contemporary studies that seek to quantify the metabolisms of specific molecules and the extent of the impact of changes in enzyme activity induced by cigarette smoke. A comprehensive evaluation of the individual associations, based on pharmacokinetic studies that also consider individual variability, is still a long way off.

Given the complexity of these interactions, a patient-centered approach is essential. This includes assessing individual smoking status, metabolic responses, and drug regimens to tailor treatment effectively. Collaborative care involving TB specialists and smoking cessation experts can enhance personalization, improve adherence, and minimize ADRs.

The overlap of high TB prevalence and smoking rates, especially in LMICs, highlights the need for targeted public health interventions. Policies that integrate smoking cessation into TB control programs could significantly reduce the global TB burden. Routine incorporation of smoking cessation into TB care has the potential to enhance treatment adherence, reduce drug resistance, and improve patient outcomes. Global frameworks already exist but are often implemented in silos. The WHO’s MPOWER package offers a set of evidence-based tobacco control measures (Monitor tobacco use, Protect people from smoke, Offer help to quit, Warn about dangers, Enforce bans on advertising, and Raise taxes) that can be synergistically aligned with the WHO’s End TB Strategy. For example, raising tobacco taxes not only reduces smoking prevalence but can also generate revenue to fund TB control programs. Similarly, integrating “Offer help to quit” services directly into TB clinics is a high-impact intervention. However, policy implementation in LMICs faces substantial barriers, including limited resources, and weak health systems. Successful country models, such as integrating brief tobacco interventions into national TB programs, demonstrate that these challenges can be overcome with political commitment and tailored strategies (WHO Report on the Global Tobacco Epidemic, 2019; The End TB Strategy, 2025; A WHO the Union Monograph on TB and Tobacco Control, 2005).

Despite these promising directions, significant knowledge gaps remain. Understanding the long-term effects of smoking cessation on drug metabolism and TB outcomes requires further investigation. Future research should focus on elucidating the specific mechanisms of DDIs, including the roles of PAHs and nicotine in enzyme modulation. Clinical trials are needed to develop optimal dosing strategies during smoking cessation for TB patients, particularly in relation to newer antituberculous drugs. Additionally, the influence of genetic polymorphisms on enzyme activity among TB smokers and quitters warrants exploration.

A concerted effort to integrate smoking cessation with TB care offers an opportunity to optimize treatment outcomes, reduce healthcare burdens, and enhance the quality of life for affected populations. These efforts should be prioritized in public health strategies to combat the dual burden of TB and tobacco use effectively.

## References

[B1] Abal A. T. Jayakrishnan B. Parwer S. El Shamy A. Abahussain E. Sharma P. N. (2005). Effect of cigarette smoking on sputum smear conversion in adults with active pulmonary tuberculosis. Respir. Med. 99 (4), 415–420. 10.1016/j.rmed.2004.08.016 15763447

[B2] Abulfathi A. A. Decloedt E. H. Svensson E. M. Diacon A. H. Donald P. Reuter H. (2019). Clinical pharmacokinetics and pharmacodynamics of rifampicin in human tuberculosis. Clin. Pharmacokinet. 58 (9), 1103–1129. 10.1007/s40262-019-00764-2 31049868

[B3] A Guide for Tuberculosis Patients to Quit Smoking (2025). A Guide for tuberculosis patients to quit smoking. Available online at: https://www.who.int/publications/i/item/9241506922 (Accessed November 29, 2024).

[B4] Ahmad N. Ahuja S. D. Akkerman O. W. Alffenaar J.-W. C. Anderson L. F. Baghaei P. (2018). Treatment correlates of successful outcomes in pulmonary multidrug-resistant tuberculosis: an individual patient data meta-analysis. Lancet London, Engl. 392 (10150), 821–834. 10.1016/S0140-6736(18)31644-1 PMC6463280 30215381

[B5] AIFA - Ricerca Farmaco. (2024). AIFA - Ricerca Farmaco. Available online at: https://medicinali.aifa.gov.it/it/#/it/dettaglio/0000022408 (Accessed November 25, 2024).

[B6] Alsayed S. S. R. Gunosewoyo H. (2023). Tuberculosis: pathogenesis, current treatment regimens and new drug targets. Int. J. Mol. Sci. 24 (6), 5202. 10.3390/ijms24065202 36982277 PMC10049048

[B7] Altet N. Latorre I. Jiménez-Fuentes M. Á. Maldonado J. Molina I. González-Díaz Y. (2017). Assessment of the influence of direct tobacco smoke on infection and active TB management. PloS One 12 (8), e0182998. 10.1371/journal.pone.0182998 28837570 PMC5570217

[B8] Amere G. A. Nayak P. Salindri A. D. Narayan K. M. V. Magee M. J. (2018). Contribution of smoking to tuberculosis incidence and mortality in high-tuberculosis-burden countries. Am. J. Epidemiol. 187 (9), 1846–1855. 10.1093/aje/kwy081 29635332 PMC6888026

[B9] American Medical Association (1992). “Division of drugs and toxicology,” in Drug evaluations annual 1994. American Medical Association.

[B10] Amin A. G. Goude R. Shi L. Zhang J. Chatterjee D. Parish T. (2008). EmbA is an essential arabinosyltransferase in Mycobacterium tuberculosis. Microbiol. Read. Engl. 154 (Pt 1), 240–248. 10.1099/mic.0.2007/012153-0 PMC2885622 18174142

[B11] Anandatheerthavarada H. K. Williams J. F. Wecker L. (1993a). Differential effect of chronic nicotine administration on brain cytochrome P4501a1/2 and P4502E1. Biochem. Biophysical Res. Commun. 194 (1), 312–318. 10.1006/bbrc.1993.1821 8333846

[B12] Anandatheerthavarada H. K. Williams J. F. Wecker L. (1993b). The chronic administration of nicotine induces cytochrome P450 in rat brain. J. Neurochem. 60 (5), 1941–1944. 10.1111/j.1471-4159.1993.tb13424.x 8473908

[B13] Arcavi L. Benowitz N. L. (2004). Cigarette smoking and infection. Archives Intern. Med. 164 (20), 2206–2216. 10.1001/archinte.164.20.2206 15534156

[B14] Aridgides D. S. Mellinger D. L. Armstrong D. A. Hazlett H. F. Dessaint J. A. Hampton T. H. (2019). Functional and metabolic impairment in cigarette smoke-exposed macrophages is tied to oxidative stress. Sci. Rep. 9 (1), 9624. 10.1038/s41598-019-46045-7 31270372 PMC6610132

[B15] Arikayce (2023). U. S. Food Drug Adm. (FDA). Available online at: https://www.accessdata.fda.gov/drugsatfda_docs/label/2023/207356s012lbl.pdf.

[B16] Aryanpur M. Hosseini M. Reza Masjedi M. Mortaz E. Tabarsi P. Hamid S. (2016). A randomized controlled trial of smoking cessation methods in patients newly-diagnosed with pulmonary tuberculosis. BMC Infect. Dis. 16, 369. 10.1186/s12879-016-1727-4 27496096 PMC4974814

[B17] Avelox (2016). U. S. Food Drug Adm. (FDA). Available online at: https://www.accessdata.fda.gov/drugsatfda_docs/label/2016/021085s063lbl.pdf.

[B18] Awaisu A. Mohamed M. H. N. Noordin N. M. Aziz N. A. Sulaiman S. A. S. Muttalif A. R. (2011). The SCIDOTS project: evidence of benefits of an integrated tobacco cessation intervention in tuberculosis care on treatment outcomes. Subst. Abuse Treat. Prev. Policy 6, 26. 10.1186/1747-597X-6-26 21943384 PMC3196696

[B19] A WHO the Union Monograph on TB and Tobacco Control (2005). Joining efforts to control two related global epidemics. Available online at: https://www.who.int/publications/i/item/WHO-HTM-TB-2007.390 (Accessed June 19, 2025).

[B20] Banerjee D. K. Ellard G. A. Gammon P. T. Waters M. F. (1974). Some observations on the pharmacology of clofazimine (B663). Am. J. Trop. Med. Hyg. 23 (6), 1110–1115. 10.4269/ajtmh.1974.23.1110 4429180

[B21] Bardou F. Raynaud C. Ramos C. Lanéelle M. A. Lanŕelle G. (1998). Mechanism of isoniazid uptake in Mycobacterium tuberculosis. Microbiol. Read. Engl. 144, 2539–2544. 10.1099/00221287-144-9-2539 9782502

[B22] Barry V. C. Belton J. G. O’Sullivan J. F. Twomey D. (1956). 650. The oxidation of derivatives of o-phenylenediamine. Part IV. A new series of glyoxalinophenazines derived from anilinoaposafranines and their behaviour on hydrogenation. J. Chem. Soc. 3347–3350. 10.1039/JR9560003347

[B23] Bates M. N. Khalakdina A. Pai M. Chang L. Lessa F. Smith K. R. (2007). Risk of tuberculosis from exposure to tobacco smoke: a systematic review and meta-analysis. Archives Intern. Med. 167 (4), 335–342. 10.1001/archinte.167.4.335 17325294

[B24] Batista L. d’Arc J. Rodrigues L. C. (2008). Smoking increases the risk of relapse after successful tuberculosis treatment. Int. J. Epidemiol. 37 (4), 841–851. 10.1093/ije/dyn113 18556729 PMC2483312

[B25] Bay J. G. Patsche C. B. Marie Svendsen N. Gomes V. F. Rudolf F. Wejse C. (2022). Tobacco smoking impact on tuberculosis treatment outcome: an observational study from west Africa. Int. J. Infect. Dis. IJID Official Publ. Int. Soc. Infect. Dis. 124 (Suppl. 1), S50–S55. 10.1016/j.ijid.2022.07.067 35914683

[B26] Belkaid Y. Hand T. W. (2014). Role of the microbiota in immunity and inflammation. Cell. 157 (1), 121–141. 10.1016/j.cell.2014.03.011 24679531 PMC4056765

[B27] Bellanca C. M. Augello E. Di Benedetto G. Burgaletto C. Flavia Cantone A. Cantarella G. (2024). A web-based scoping review assessing the influence of smoking and smoking cessation on antidiabetic drug meabolism: implications for medication efficacy. Front. Pharmacol. 15, 1406860. 10.3389/fphar.2024.1406860 38957391 PMC11217182

[B28] Benowitz N. L. (1997). The role of nicotine in smoking-related cardiovascular disease. Prev. Med. 26 (4), 412–417. 10.1006/pmed.1997.0175 9245658

[B29] Benowitz N. L. Jacob P. (1993). Nicotine and cotinine elimination pharmacokinetics in smokers and nonsmokers. Clin. Pharmacol. Ther. 53 (3), 316–323. 10.1038/clpt.1993.27 8453850

[B30] Benowitz N. L. Jacob P. (2000). Effects of cigarette smoking and carbon monoxide on nicotine and cotinine metabolism. Clin. Pharmacol. Ther. 67 (6), 653–659. 10.1067/mcp.2000.107086 10872647

[B31] Benowitz N. L. Peng M. Jacob P. (2003). Effects of cigarette smoking and carbon monoxide on chlorzoxazone and caffeine metabolism. Clin. Pharmacol. Ther. 74 (5), 468–474. 10.1016/j.clpt.2003.07.001 14586387

[B32] Bergen A. W. Javitz H. S. Krasnow R. Michel M. Nishita D. Conti D. V. (2014). Organic cation transporter variation and response to smoking cessation therapies. Nicotine and Tob. Res. Official J. Soc. Res. Nicotine Tob. 16 (12), 1638–1646. 10.1093/ntr/ntu161 PMC4296186 25143296

[B34] Blaschke T. F. Skinner M. H. (1996). The clinical pharmacokinetics of rifabutin. Clin. Infect. Dis. An Official Publ. Infect. Dis. Soc. Am. 22, S15–S22. 10.1093/clinids/22.supplement_1.s15 8785251

[B35] Bloom B. R. Atun R. Cohen T. Dye C. Fraser H. Gomez G. B. (2017). “Tuberculosis,” in Major infectious diseases. Editors Holmes K. K. Bertozzi S. Bloom B. R. Jha P., 3rd ed. (Washington (DC): The International Bank for Reconstruction and Development/The World Bank). Available online at: http://www.ncbi.nlm.nih.gov/books/NBK525174/. 30212055

[B36] Borovinskaya M. A. Pai R. D. Zhang W. Schuwirth B. S. Holton J. M. Hirokawa Go (2007). Structural basis for aminoglycoside inhibition of bacterial ribosome recycling. Nat. Struct. and Mol. Biol. 14 (8), 727–732. 10.1038/nsmb1271 17660832

[B37] Boshoff H. I. Mizrahi V. Barry C. E. (2002). Effects of pyrazinamide on fatty acid synthesis by whole mycobacterial cells and purified fatty acid synthase I. J. Bacteriol. 184 (8), 2167–2172. 10.1128/JB.184.8.2167-2172.2002 11914348 PMC134955

[B38] Branch R. A. Adedoyin A. Frye R. F. Wilson J. W. Romkes M. (2000). *In vivo* modulation of CYP enzymes by quinidine and rifampin. Clin. Pharmacol. Ther. 68 (4), 401–411. 10.1067/mcp.2000.110561 11061580

[B39] British National Formulary (BNF) (2015). British national formulary (BNF). Pharmaceutical Press. Available online at: https://www.pharmaceuticalpress.com/products/british-national-formulary/ (Accessed March 12, 2024).

[B40] Bulitta J. B. Ly N. S. Landersdorfer C. B. Wanigaratne N. A. Velkov T. Yadav R. (2015). Two mechanisms of killing of Pseudomonas aeruginosa by tobramycin assessed at multiple inocula via mechanism-based modeling. Antimicrob. Agents Chemother. 59 (4), 2315–2327. 10.1128/AAC.04099-14 25645838 PMC4356757

[B41] Cahill K. Stevens S. Perera R. Lancaster T. (2013). Pharmacological interventions for smoking cessation: an overview and network meta‐analysis. Cochrane Database Syst. Rev. 2013 (5), CD009329. 10.1002/14651858.CD009329.pub2 23728690 PMC8406789

[B42] Capastat (2019). U. S. Food Drug Adm. (FDA). Available online at: https://www.accessdata.fda.gov/drugsatfda_docs/label/2019/050095s072lbl.pdf.

[B43] Champix (2006). Champix. European Medicines Agency EMA. Available online at: https://www.ema.europa.eu/en/medicines/human/EPAR/champix.

[B44] Chandra Acharya P. Kurosu M. (2023). Medicinal chemistry of chemotherapeutic agents: a comprehensive resource of anti-infective and anti-cancer drugs. 1st edition. Academic Press. 10.1016/C2020-0-03892-2

[B45] Chen H. Cowan M. J. Hasday J. D. Vogel S. N. Medvedev A. E. (2007). Tobacco smoking inhibits expression of proinflammatory cytokines and activation of IL-1R-associated kinase, P38, and NF-kappaB in alveolar macrophages stimulated with TLR2 and TLR4 agonists. J. Immunol. Baltim. Md 179, 6097–6106. 10.4049/jimmunol.179.9.6097 17947684

[B46] Cholo M. C. Mothiba M. T. Fourie B. Anderson R. (2017). Mechanisms of action and therapeutic efficacies of the lipophilic antimycobacterial agents clofazimine and bedaquiline. J. Antimicrob. Chemother. 72 (2), 338–353. 10.1093/jac/dkw426 27798208

[B47] Cohen G. Bellanca C. M. Bernardini R. Rose J. E. Polosa R. (2024). Personalized and adaptive interventions for smoking cessation: emerging trends and determinants of efficacy. iScience 27 (11), 111090. 10.1016/j.isci.2024.111090 39620129 PMC11607540

[B48] Collier A. C. Tingle M. D. Paxton J. W. Mitchell M. D. Keelan J. A. (2002). Metabolizing enzyme localization and activities in the first trimester human placenta: the effect of maternal and gestational age, smoking and alcohol consumption. Hum. Reprod. Oxf. Engl. 17 (10), 2564–2572. 10.1093/humrep/17.10.2564 12351530

[B49] Connarn J. N. Zhang X. Babiskin A. Sun D. (2015). Metabolism of bupropion by carbonyl reductases in liver and intestine. Drug Metabolism Dispos. Biol. Fate Chem. 43 (7), 1019–1027. 10.1124/dmd.115.063107 PMC6067387 25904761

[B50] Conradie F. Bagdasaryan T. R. Borisov S. Howell P. Mikiashvili L. Ngubane N. (2022). Bedaquiline-pretomanid-linezolid regimens for drug-resistant tuberculosis. N. Engl. J. Med. 387 (9), 810–823. 10.1056/NEJMoa2119430 36053506 PMC9490302

[B51] Conradie F. Diacon A. H. Ngubane N. Howell P. Everitt D. Crook A. M. (2020). Treatment of highly drug-resistant pulmonary tuberculosis. N. Engl. J. Med. 382 (10), 893–902. 10.1056/NEJMoa1901814 32130813 PMC6955640

[B52] Corleis B. Tzouanas C. N. Wadsworth M. H. Cho J. L. Linder A. H. Schiff A. E. (2023). Tobacco smoke exposure recruits inflammatory airspace monocytes that establish permissive lung niches for Mycobacterium tuberculosis. Sci. Transl. Med. 15 (725), eadg3451. 10.1126/scitranslmed.adg3451 38055798 PMC12289333

[B53] Court M. H. (2010). Interindividual variability in hepatic drug glucuronidation: studies into the role of age, sex, enzyme inducers, and genetic polymorphism using the human liver bank as a model system. Drug Metab. Rev. 42 (1), 209–224. 10.3109/03602530903209288 19821798 PMC6174030

[B54] Crabol Y. Catherinot E. Veziris N. Vincent J. Olivier L. (2016). Rifabutin: where do we stand in 2016? J. Antimicrob. Chemother. 71 (7), 1759–1771. 10.1093/jac/dkw024 27009031

[B55] Dalet-Beluche I. Boulenc X. Fabre G. Maurel P. Bonfils C. (1992). Purification of two cytochrome P450 isozymes related to CYP2A and CYP3A gene families from monkey (baboon, Papio Papio) liver microsomes. Cross reactivity with human forms. Eur. J. Biochem. 204 (2), 641–648. 10.1111/j.1432-1033.1992.tb16677.x 1541278

[B56] Dallenga T. Repnik U. Corleis B. Eich J. Reimer R. Griffiths G. W. (2017). Tuberculosis-induced necrosis of infected neutrophils promotes bacterial growth following phagocytosis by macrophages. Cell. Host and Microbe 22 (4), 519–530.e3. 10.1016/j.chom.2017.09.003 29024644

[B57] Dana N. R. Rika S. Pudia M I. Alexander M. B. Muthia S. Linda R. (2024). Modifiable and non-modifiable risk factors for tuberculosis among adults in Indonesia: a systematic review and meta-analysis. Afr. J. Infect. Dis. 18 (2), 19–28. 10.21010/Ajidv18i2.3 38606192 PMC11004781

[B58] Delaney J. Timbrell J. A. (1995). Role of cytochrome P450 in hydrazine toxicity in isolated hepatocytes *in vitro* . Xenobiotica; Fate Foreign Compd. Biol. Syst. 25 (12), 1399–1410. 10.3109/00498259509061927 8719914

[B59] Deltyba (2014). Deltyba. European Medicines Agency EMA. Available online at: https://www.ema.europa.eu/en/medicines/human/EPAR/deltyba.

[B60] Dogar O. Keding A. Gabe R. Marshall A.-M. Huque R. Barua D. (2020). Cytisine for smoking cessation in patients with tuberculosis: a multicentre, randomised, double-blind, placebo-controlled phase 3 trial. Lancet 8 (11), e1408–e1417. 10.1016/S2214-109X(20)30312-0 33069301

[B61] Dover L. G. Alahari A. Paul G. Gomes J. M. Bhowruth V. Reynolds R. C. (2007). EthA, a common activator of thiocarbamide-containing drugs acting on different mycobacterial targets. Antimicrob. Agents Chemother. 51 (3), 1055–1063. 10.1128/AAC.01063-06 17220416 PMC1803108

[B62] Dragacci S. Hamar-Hansen C. Fournel-Gigleux S. Lafaurie C. Magdalou J. Siest G. (1987). Comparative study of clofibric acid and bilirubin glucuronidation in human liver microsomes. Biochem. Pharmacol. 36 (22), 3923–3927. 10.1016/0006-2952(87)90459-x 3120730

[B63] DrugBank Clinical API Plugins (2024). DrugBank clinical API Plugins. Available online at: https://dev.drugbank.com/demo/ddi_checker (Accessed March 12, 2024).

[B64] Drug Interaction Checker Quickly Check Your Meds (2024). Drug interaction checker quickly Check Your Meds. Drugs. Com. Available online at: https://www.drugs.com/drug_interactions.html (Accessed November 26, 2024).

[B65] Drug Interactions Checker - Medscape Drug Reference Database (2024). Drug interactions checker - Medscape drug reference database. Available online at: https://reference.medscape.com/drug-interactionchecker (Accessed November 26, 2024).

[B66] DRUG-REAX (2024). University of technology sydney. Available online at: https://search.lib.uts.edu.au/discovery/fulldisplay/alma991001040579705671/61UTS_INST:61UTS (Accessed March 12, 2024).

[B67] Drug-Resistant Tuberculosis (2024). Key updates to the treatment of drug-resistant tuberculosis: rapid communication. Available online at: https://www.who.int/publications/i/item/B09123 (Accessed November 25, 2024).

[B68] Dryden M. S. (2011). Linezolid pharmacokinetics and pharmacodynamics in clinical treatment. J. Antimicrob. Chemother. 66, 7–15. 10.1093/jac/dkr072 21521707

[B69] Ellard G. A. Gammon P. T. (1976). Pharmacokinetics of isoniazid metabolism in man. J. Pharmacokinet. Biopharm. 4 (2), 83–113. 10.1007/BF01086149 950592

[B70] Faber M. S. Uwe F. (2004). Time response of cytochrome P450 1A2 activity on cessation of heavy smoking. Clin. Pharmacol. and Ther. 76 (2), 178–184. 10.1016/j.clpt.2004.04.003 15289794

[B71] Fàbrega A. Madurga S. Giralt E. Vila J. (2009). Mechanism of action of and resistance to quinolones. Microb. Biotechnol. 2 (1), 40–61. 10.1111/j.1751-7915.2008.00063.x 21261881 PMC3815421

[B72] Fan J. Yuan Z. Ran M. Tang J. Zhu J. Aldrich M. C. (2023). Cross-talks between gut microbiota and tobacco smoking: a two-sample mendelian randomization study. BMC Med. 21 (1), 163. 10.1186/s12916-023-02863-1 37118782 PMC10148467

[B73] Feldman C. Theron A. J. Cholo M. C. Anderson R. (2024). Cigarette smoking as a risk factor for tuberculosis in adults: epidemiology and aspects of disease pathogenesis. Pathog. Basel, Switz. 13 (2), 151. 10.3390/pathogens13020151 PMC10892798 38392889

[B74] Feng P. C. Fenselau C. C. Jacobson R. R. (1981). Metabolism of clofazimine in leprosy patients. Drug Metabolism Dispos. Biol. Fate Chem. 9 (6), 521–524. 10.1016/s0090-9556(25)06563-8 6120809

[B75] Feng Y. Kong Y. Barnes P. F. Huang F.-F. Klucar P. Wang X. (2011). Exposure to cigarette smoke inhibits the pulmonary T-cell response to influenza virus and Mycobacterium tuberculosis. Infect. Immun. 79 (1), 229–237. 10.1128/IAI.00709-10 20974820 PMC3019896

[B76] Fish D. N. Chow A. T. (1997). The clinical pharmacokinetics of levofloxacin. Clin. Pharmacokinet. 32 (2), 101–119. 10.2165/00003088-199732020-00002 9068926

[B77] Fuhr U. Rost K. L. (1994). Simple and reliable CYP1A2 phenotyping by the paraxanthine/caffeine ratio in plasma and in saliva. Pharmacogenetics 4 (3), 109–116. 10.1097/00008571-199406000-00001 7920690

[B78] Gajalakshmi V. Peto R. Kanaka T. S. Jha P. (2003). Smoking and mortality from tuberculosis and other diseases in India: retrospective study of 43000 adult male deaths and 35000 controls. Lancet London, Engl. 362 (9383), 507–515. 10.1016/S0140-6736(03)14109-8 12932381

[B79] Getahun H. Gunneberg C. Granich R. Paul N. (2010). HIV infection-associated tuberculosis: the epidemiology and the response. Clin. Infect. Dis. An Official Publ. Infect. Dis. Soc. Am. 50 (Suppl. 3), S201–S207. 10.1086/651492 20397949

[B80] Global Tuberculosis Report (2023). Glob. Tuberc. Rep. Available online at: https://www.who.int/teams/global-tuberculosis-programme/tb-reports/global-tuberculosis-report-2023 (Accessed 25 November 2024).

[B81] Goodman L. S. Goodman Gilman A. Hardman L. Limbird L. E. Goodman Gilman A. (2001). Goodman and gilman’s the pharmacological basis of therapeutics.

[B82] Goodwin B. Redinbo M. R. Kliewer S. A. (2002). Regulation of Cyp3a gene transcription by the pregnane x receptor. Annu. Rev. Pharmacol. Toxicol. 42, 1–23. 10.1146/annurev.pharmtox.42.111901.111051 11807162

[B83] Goude R. Amin A. G. Chatterjee D. Parish T. (2009). The arabinosyltransferase EmbC is inhibited by ethambutol in Mycobacterium tuberculosis. Antimicrob. Agents Chemother. 53 (10), 4138–4146. 10.1128/AAC.00162-09 19596878 PMC2764220

[B84] Hardman J. G. Limbird L. E. Gilman A. G. McGraw H. (2001). Goodman and gilman’s the pharmacological basis of therapeutics. 10th Edition (New York: McGraw-Hill), 1392–1393.

[B85] Hayward S. Harding R. M. McShane H. Tanner R. (2018). Factors influencing the higher incidence of tuberculosis among migrants and ethnic minorities in the UK. F1000Research 7, 461. 10.12688/f1000research.14476.2 30210785 PMC6107974

[B86] Helmig S. Udo Seelinger J. Philipp-Gehlhaar M. Döhrel J. Schneider J. (2010). Cyp1B1 mRNA expression in correlation to cotinine levels with respect to the Cyp1B1 L432V gene polymorphism. Eur. J. Epidemiol. 25 (12), 867–873. 10.1007/s10654-010-9505-x 20830506

[B87] Henderson M. C. Siddens L. K. Morré J. T. Krueger S. K. Williams D. E. (2008). Metabolism of the anti-tuberculosis drug ethionamide by mouse and human FMO1, FMO2 and FMO3 and mouse and human lung microsomes. Toxicol. Appl. Pharmacol. 233 (3), 420–427. 10.1016/j.taap.2008.09.017 18930751 PMC2626250

[B88] Hirota T. Takane H. Higuchi S. Ieiri I. (2008). Epigenetic regulation of genes encoding drug-metabolizing enzymes and transporters; DNA methylation and other mechanisms. Curr. Drug Metab. 9 (1), 34–38. 10.2174/138920008783331130 18220569

[B89] Hodge S. Hodge G. Ahern J. Jersmann H. Holmes M. Reynolds P. N. (2007). Smoking alters alveolar macrophage recognition and phagocytic ability: implications in chronic obstructive pulmonary disease. Am. J. Respir. Cell. Mol. Biol. 37 (6), 748–755. 10.1165/rcmb.2007-0025OC 17630319

[B90] Hoffmann D. H. Hoffmann I. (1997). The changing cigarette, 1950-1995. J. Toxicol. Environ. Health 50 (4), 307–364. 10.1080/009841097160393 9120872

[B91] Honda Y. Takahashi H. Kuroki Y. Akino T. Abe S. (1996). Decreased contents of surfactant proteins A and D in BAL fluids of healthy smokers. Chest 109 (4), 1006–1009. 10.1378/chest.109.4.1006 8635323

[B92] Howlader S. Kim M.-J. Jony M. R. Long N. P. Cho Y.-S. Kim D.-H. (2022). Characterization of clofazimine metabolism in human liver microsomal incubation *in vitro* . Antimicrob. Agents Chemother. 66 (10), e0056522. 10.1128/aac.00565-22 36190267 PMC9578437

[B93] Hughes D. A. Haslam P. L. Townsend P. J. Turner-Warwick M. (1985). Numerical and functional alterations in circulatory lymphocytes in cigarette smokers. Clin. Exp. Immunol. 61 (2), 459–466. 2931227 PMC1577295

[B94] Hussain Z. Zhu J. Ma X. (2021). Metabolism and hepatotoxicity of pyrazinamide, an antituberculosis drug. Drug Metabolism Dispos. Biol. Fate Chem. 49 (8), 679–682. 10.1124/dmd.121.000389 PMC8407665 34074731

[B95] INTERCheck WEB (2024). INTERCheck WEB. Available online at: https://intercheckweb.marionegri.it/home#/main (Accessed November 26, 2024).

[B96] Jamis-Dow C. A. Katki A. G. Collins J. M. Klecker R. W. (1997). Rifampin and rifabutin and their metabolism by human liver esterases. Xenobiotica; Fate Foreign Compd. Biol. Syst. 27 (10), 1015–1024. 10.1080/004982597239994 9364739

[B97] Jefferson J. W. Pradko J. F. Muir K. T. (2005). Bupropion for major depressive disorder: pharmacokinetic and formulation considerations. Clin. Ther. 27 (11), 1685–1695. 10.1016/j.clinthera.2005.11.011 16368442

[B98] Jha P. Jacob B. Gajalakshmi V. Gupta P. C. Dhingra N. Kumar R. (2008). A nationally representative case-control study of smoking and death in India. N. Engl. J. Med. 358 (11), 1137–1147. 10.1056/NEJMsa0707719 18272886

[B99] Johnson T. M. Rivera C. G. Lee G. Zeuli J. D. (2024). Pharmacology of emerging drugs for the treatment of multi-drug resistant tuberculosis. J. Clin. Tuberc. Other Mycobact. Dis. 37, 100470. 10.1016/j.jctube.2024.100470 39188351 PMC11345926

[B100] Kim M. Johnson C. E. Schmalstig A. A. Annis A. Wessel S. E. Van Horn B. (2022). A long-acting formulation of rifabutin is effective for prevention and treatment of Mycobacterium tuberculosis. Nat. Commun. 13 (1), 4455. 10.1038/s41467-022-32043-3 35941109 PMC9360445

[B101] Kirkham P. A. Spooner G. Rahman I. Rossi A. G. (2004). Macrophage phagocytosis of apoptotic neutrophils is compromised by matrix proteins modified by cigarette smoke and lipid peroxidation products. Biochem. Biophysical Res. Commun. 318 (1), 32–37. 10.1016/j.bbrc.2004.04.003 15110749

[B102] Knox C. Wilson M. Klinger C. M. Franklin M. Oler E. Wilson A. (2024). DrugBank 6.0: the DrugBank knowledgebase for 2024. Nucleic Acids Res. 52 (D1), D1265–D1275. 10.1093/nar/gkad976 37953279 PMC10767804

[B103] Kroon L. A. (2007). Drug interactions with smoking. Am. J. Health-System Pharm. AJHP Official J. Am. Soc. Health-System Pharm. 64 (18), 1917–1921. 10.2146/ajhp060414 17823102

[B104] Krueger S. K. Williams D. E. (2005). Mammalian flavin-containing monooxygenases: structure/function, genetic polymorphisms and role in drug metabolism. Pharmacol. and Ther. 106 (3), 357–387. 10.1016/j.pharmthera.2005.01.001 15922018 PMC1828602

[B105] Kumagai T. Suzuki H. Sasaki T. Sakaguchi S. Miyairi S. Yamazoe Y. (2012). Polycyclic aromatic hydrocarbons activate CYP3A4 gene transcription through human pregnane X receptor. Drug Metabolism Pharmacokinet. 27 (2), 200–206. 10.2133/dmpk.dmpk-11-rg-094 22076448

[B106] Lacroix C. Hoang T. P. Nouveau J. Guyonnaud C. Laine G. Duwoos H. (1989). Pharmacokinetics of pyrazinamide and its metabolites in healthy subjects. Eur. J. Clin. Pharmacol. 36 (4), 395–400. 10.1007/BF00558302 2737233

[B107] Lamprene (2019). U. S. Food Drug Adm. (FDA). Available online at: https://www.accessdata.fda.gov/drugsatfda_docs/label/2019/019500s014lbl.pdf.

[B108] Leach K. L. Brickner S. J. Noe M. C. Miller P. F. (2011). Linezolid, the first oxazolidinone antibacterial agent. Ann. N. Y. Acad. Sci. 1222, 49–54. 10.1111/j.1749-6632.2011.05962.x 21434942

[B109] Lechartier B. Cole S. T. (2015). Mode of action of clofazimine and combination therapy with benzothiazinones against Mycobacterium tuberculosis. Antimicrob. Agents Chemother. 59 (8), 4457–4463. 10.1128/AAC.00395-15 25987624 PMC4505229

[B110] Lee B. L. Benowitz N. L. Jacob P. (1987). Influence of tobacco abstinence on the disposition kinetics and effects of nicotine. Clin. Pharmacol. Ther. 41 (4), 474–479. 10.1038/clpt.1987.59 3829584

[B111] Lee S. Y. Jang H. Lee J.-Y. Kwon K. Oh S. J. Kim S. K. (2014). Inhibition of cytochrome P450 by ethambutol in human liver microsomes. Toxicol. Lett. 229 (1), 33–40. 10.1016/j.toxlet.2014.06.006 24910189

[B112] Leemans J. C. Thepen T. Weijer S. Florquin S. van Rooijen N. van de Winkel J. G. (2005). Macrophages play a dual role during pulmonary tuberculosis in mice. J. Infect. Dis. 191 (1), 65–74. 10.1086/426395 15593005

[B113] Lehmann J. M. McKee D. D. Watson M. A. Willson T. M. Moore J. T. Kliewer S. A. (1998). The human orphan nuclear receptor PXR is activated by compounds that regulate CYP3A4 gene expression and cause drug interactions. J. Clin. Investigation 102 (5), 1016–1023. 10.1172/JCI3703 PMC508967 9727070

[B115] Leite G. Barlow G. M. Hosseini A. Parodi G. Pimentel M. L. Wang J. (2022). Smoking has disruptive effects on the small bowel luminal microbiome. Sci. Rep. 12 (1), 6231. 10.1038/s41598-022-10132-z 35422064 PMC9010470

[B116] Les interactions médicamenteuses (2024). “Les interactions médicamenteuses,” in Vidal. Available online at: https://www.vidal.fr/medicaments/utilisation/prendre-traitement/interactions-medicamenteuses.html (Accessed March 12, 2024).

[B117] Levaquin (2008). U. S. Food Drug Adm. (FDA). Available online at: https://www.accessdata.fda.gov/drugsatfda_docs/label/2008/021721s020_020635s57_020634s52_lbl.pdf.

[B118] Levy L. (1974). Pharmacologic studies of clofazimine. Am. J. Trop. Med. Hyg. 23 (6), 1097–1109. 10.4269/ajtmh.1974.23.1097 4611255

[B119] Lewis J. M. Sloan D. J. (2015). The role of delamanid in the treatment of drug-resistant tuberculosis. Ther. Clin. Risk Manag. 11, 779–791. 10.2147/TCRM.S71076 25999726 PMC4437614

[B120] Lips K. S. Volk C. Schmitt B. M. Pfeil U. Arndt P. Miska D. (2005). Polyspecific cation transporters mediate luminal Release of acetylcholine from bronchial epithelium. Am. J. Respir. Cell. Mol. Biol. 33 (1), 79–88. 10.1165/rcmb.2004-0363OC 15817714

[B121] Lönnroth K. Jaramillo E. Williams B. G. Dye C. Raviglione M. (2009). Drivers of tuberculosis epidemics: the role of risk factors and social determinants. Soc. Sci. and Med. 68 (12), 2240–2246. 10.1016/j.socscimed.2009.03.041 19394122

[B122] Loos U. Musch E. Jensen J. C. Schwabe H. K. Eichelbaum M. (1987). Influence of the enzyme induction by rifampicin on its presystemic metabolism. Pharmacol. and Ther. 33 (1), 201–204. 10.1016/0163-7258(87)90052-0 3628475

[B123] Lugg S. T. Scott A. Parekh D. Naidu B. Thickett D. R. (2022). Cigarette smoke exposure and alveolar macrophages: mechanisms for lung disease. Thorax 77 (1), 94–101. 10.1136/thoraxjnl-2020-216296 33986144 PMC8685655

[B124] Maartens G. Brill M. J. E. Pandie M. Svensson E. M. (2018). Pharmacokinetic interaction between bedaquiline and clofazimine in patients with drug-resistant tuberculosis. Int. J. Tuberc. Lung Dis. Official J. Int. Union Against Tuberc. Lung Dis. 22 (1), 26–29. 10.5588/ijtld.17.0615 29145924

[B125] Mahishale V. Patil B. Lolly M. Eti A. Khan S. (2015). Prevalence of smoking and its impact on treatment outcomes in newly diagnosed pulmonary tuberculosis patients: a hospital-based prospective study. Chonnam Med. J. 51 (2), 86–90. 10.4068/cmj.2015.51.2.86 26306303 PMC4543154

[B126] Maideen N. M. P. (2019). Tobacco smoking and its drug interactions with comedications involving CYP and UGT enzymes and nicotine. World J. Pharmacol. 8 (2), 14–25. 10.5497/wjp.v8.i2.14

[B127] Malo A. Kellermann T. Ignatius E. H. Dooley K. E. Dawson R. Joubert A. (2021). A validated liquid chromatography tandem mass spectrometry assay for the analysis of pretomanid in plasma samples from pulmonary tuberculosis patients. J. Pharm. Biomed. Analysis 195, 113885. 10.1016/j.jpba.2020.113885 PMC7868581 33406472

[B128] McDonnell A. M. Dang C. H. (2013). Basic review of the cytochrome P450 system. J. Adv. Pract. Oncol. 4 (4), 263–268. 10.6004/jadpro.2013.4.4.7 25032007 PMC4093435

[B129] McKENNIS H. Yard A. S. Weatherby J. H. Hagy J. A. (1959). Acetylation of hydrazine and the formation of 1,2-diacetylhydrazine *in vivo* . J. Pharmacol. Exp. Ther. 126 (2), 109–116. 10.1016/s0022-3565(25)25600-2 13665515

[B130] McNab F. W. Berry M. P. R. Graham C. M. Bloch S. A. A. Oni T. Wilkinson K. A. (2011). Programmed death ligand 1 is over-expressed by neutrophils in the blood of patients with active tuberculosis. Eur. J. Immunol. 41 (7), 1941–1947. 10.1002/eji.201141421 21509782 PMC3179592

[B131] Meech R. Mackenzie P. I. (1997). Structure and function of uridine diphosphate glucuronosyltransferases. Clin. Exp. Pharmacol. Physiology 24 (12), 907–915. 10.1111/j.1440-1681.1997.tb02718.x 9406655

[B132] Mesens N. Steemans M. Hansen E. Verheyen G. R. Van Goethem F. Van Gompel J. (2010). Screening for phospholipidosis induced by central nervous drugs: comparing the predictivity of an *in vitro* assay to high throughput *in silico* Assays. Toxicol. Vitro An Int. J. Publ. Assoc. BIBRA 24 (5), 1417–1425. 10.1016/j.tiv.2010.04.007 20430096

[B133] Metushi I. G. Jack U. (2014). Isoniazid-induced liver injury and immune response in mice. J. Immunotoxicol. 11 (4), 383–392. 10.3109/1547691X.2013.860644 24303880

[B134] Miller L. G. Goldstein G. Murphy M. Ginns L. C. (1982). Reversible alterations in immunoregulatory T cells in smoking. Analysis by monoclonal antibodies and flow cytometry. Chest 82 (5), 526–529. 10.1378/chest.82.5.526 6982152

[B135] Mitchison J. M. (1956). The thickness of the cortex of the sea-urchin egg and the problem of the vitelline membrane. J. Cell. Sci. s3-97 (37), 109–121. 10.1242/jcs.s3-97.37.109

[B136] Mitnick C. D. Bryan M. G. Peloquin C. A. (2009). Tuberculosis pharmacotherapy: strategies to optimize patient care. Expert Opin. Pharmacother. 10 (3), 381–401. 10.1517/14656560802694564 19191677 PMC2674232

[B137] Moré J. M. Voelker D. R. Silveira L. J. Edwards M. G. Chan E. D. Bowler R. P. (2010). Smoking reduces surfactant protein D and phospholipids in patients with and without chronic obstructive pulmonary disease. BMC Pulm. Med. 10, 53. 10.1186/1471-2466-10-53 20973980 PMC2987951

[B138] Myambutol (2008). U. S. Food Drug Adm. (FDA). Available online at: https://www.accessdata.fda.gov/drugsatfda_docs/label/2008/016320s063lbl.pdf.

[B139] Nakajima A. Fukami T. Kobayashi Y. Watanabe A. Nakajima M. Yokoi T. (2011). Human arylacetamide deacetylase is responsible for deacetylation of rifamycins: rifampicin, rifabutin, and rifapentine. Biochem. Pharmacol. 82 (11), 1747–1756. 10.1016/j.bcp.2011.08.003 21856291

[B140] National Center for Chronic Disease Prevention and Health Promotion (US) Office on Smoking and Health (2014). *The health Consequences of smoking—50 Years of progress: a Report of the surgeon general*. Reports of the surgeon general. Atlanta (GA): Centers for Disease Control and Prevention US. Available online at: http://www.ncbi.nlm.nih.gov/books/NBK179276/. 24455788

[B141] Ngo S. C. Zimhony O. Chung W. J. Sayahi H. Jacobs W. R. Welch J. T. (2007). Inhibition of isolated Mycobacterium tuberculosis fatty acid synthase I by pyrazinamide analogs. Antimicrob. Agents Chemother. 51 (7), 2430–2435. 10.1128/AAC.01458-06 17485499 PMC1913273

[B142] Niemi M. Backman J. T. Fromm M. F. Neuvonen P. J. Kivistö K. T. (2003). Pharmacokinetic interactions with rifampicin: clinical relevance. Clin. Pharmacokinet. 42 (9), 819–850. 10.2165/00003088-200342090-00003 12882588

[B143] Nijenbandring de Boer R. Cobelens F. Ramalho D. Miranda P. F. C. Logo K. Oliveira H. (2014). Delayed culture conversion due to cigarette smoking in active pulmonary tuberculosis patients. Tuberc. Edinb. Scotl. 94 (1), 87–91. 10.1016/j.tube.2013.10.005 24321739

[B144] Novotny T. E. (2008). Smoking cessation and tuberculosis: connecting the DOTS. Int. J. Tuberc. Lung Dis. Official J. Int. Union Against Tuberc. Lung Dis. 12 (10), 1103. 18812035

[B145] Noykhovich E. Mookherji S. Roess A. (2019). The risk of tuberculosis among populations living in slum settings: a systematic review and meta-analysis. J. Urban Health Bull. N. Y. Acad. Med. 96 (2), 262–275. 10.1007/s11524-018-0319-6 PMC6458189 30341562

[B146] Nunn A. J. Phillips P. P. J. Meredith S. K. Chiang C.-Y. Conradie F. Dalai D. (2019). A trial of a shorter regimen for rifampin-resistant tuberculosis. N. Engl. J. Med. 380 (13), 1201–1213. 10.1056/NEJMoa1811867 30865791

[B231] Nyang'wa B. T. Berry C. Kazounis E. Motta I. Parpieva N. Tigay Z. (2022). A 24-week, all-oral regimen for rifampin-resistant tuberculosis. N. Engl. J. Med. 387 (25), 2331–2343. 10.1056/NEJMoa2117166 36546625

[B147] O’Leary S. M. Coleman M. M. Mei Chew W. Morrow C. McLaughlin A. M. Gleeson L. E. (2014). Cigarette smoking impairs human pulmonary immunity to Mycobacterium tuberculosis. Am. J. Respir. Crit. Care Med. 190 (12), 1430–1436. 10.1164/rccm.201407-1385OC 25390734

[B148] O’Malley M. King A. N. Conte M. Ellingrod V. L. Ramnath N. (2014). Effects of cigarette smoking on metabolism and effectiveness of systemic therapy for lung cancer. J. Thorac. Oncol. 9 (7), 917–926. 10.1097/JTO.0000000000000191 24926542

[B149] Pachauri V. Flora S. J. S. (2013). Effect of nicotine pretreatment on arsenic-induced oxidative stress in male wistar rats. Hum. and Exp. Toxicol. 32 (9), 972–982. 10.1177/0960327112474833 23475432

[B150] Pampaloni A. Locatelli M. E. Venanzi Rullo E. Alaimo S. Cosentino F. Marino A. (2021). “Diagnosis on the dock” project: a proactive screening program for diagnosing pulmonary tuberculosis in disembarking refugees and new sei model. Int. J. Infect. Dis. 106, 98–104. 10.1016/j.ijid.2021.03.032 33737130

[B151] Pareek M. Greenaway C. Noori T. Munoz J. Zenner D. (2016). The impact of migration on tuberculosis epidemiology and control in high-income countries: a review. BMC Med. 14 (1), 48. 10.1186/s12916-016-0595-5 27004556 PMC4804514

[B152] Peets E. A. Sweeney W. M. Place V. A. Buyske D. A. (1965). The absorption, excretion, and metabolic fate of ethambutol in man. Am. Rev. Respir. Dis. 91, 51–58. 10.1164/arrd.1965.91.1.51 14260000

[B153] PharmGKB (2024). PharmGKB. Available online at: https://www.pharmgkb.org/(Accessed November 27, 2024).

[B154] Pienaar E. Sarathy J. Prideaux B. Dietzold J. Dartois V. Kirschner D. E. (2017). Comparing efficacies of moxifloxacin, levofloxacin and gatifloxacin in tuberculosis granulomas using a multi-scale systems pharmacology approach. PLoS Comput. Biol. 13 (8), e1005650. 10.1371/journal.pcbi.1005650 28817561 PMC5560534

[B155] Polosa R. Benowitz N. L. (2011). Treatment of nicotine addiction: present therapeutic options and pipeline developments. Trends Pharmacol. Sci. 32 (5), 281–289. 10.1016/j.tips.2010.12.008 21256603 PMC5564372

[B156] Pretomanid (2019). U. S. Food Drug Adm. (FDA). Available online at: https://www.accessdata.fda.gov/drugsatfda_docs/label/2019/212862s000lbl.pdf.

[B157] Priftin (2000). U. S. Food Drug Adm. (FDA). Available online at: https://www.accessdata.fda.gov/drugsatfda_docs/nda/2000/21024S5_Priftin.cfm.

[B158] Pstragowski M. Zbrzezna M. Bujalska-Zadrozny M. (2017). Advances in pharmacotherapy of tuberculosiS. Acta Pol. Pharm. 74 (1), 3–11. 29474756

[B159] Quan D. H. Kwong A. J. Hansbro P. M. Britton W. J. (2022). No smoke without fire: the impact of cigarette smoking on the immune control of tuberculosis. Eur. Respir. Rev. An Official J. Eur. Respir. Soc. 31 (164), 210252. 10.1183/16000617.0252-2021 PMC9488690 35675921

[B160] Rae J. M. Johnson M. D. Lippman M. E. Flockhart D. A. (2001). Rifampin is a selective, pleiotropic inducer of drug metabolism genes in human hepatocytes: studies with cDNA and oligonucleotide expression arrays. J. Pharmacol. Exp. Ther. 299 (3), 849–857. 10.1016/s0022-3565(24)29202-8 11714868

[B161] Rawat A. Chaturvedi S. Singh A. K. Guleria A. Dubey D. Keshari A. K. (2018). Metabolomics approach discriminates toxicity index of pyrazinamide and its metabolic products, pyrazinoic acid and 5-hydroxy pyrazinoic acid. Hum. and Exp. Toxicol. 37 (4), 373–389. 10.1177/0960327117705426 28425350

[B162] Riccardi N. Monari C. Antonello R. M. Saderi L. Occhineri S. Pontarelli A. (2024). TB outpatient care in a high-income, low-incidence country. Int. J. Tuberc. Lung Dis. Official J. Int. Union Against Tuberc. Lung Dis. 28 (10), 513–515. 10.5588/ijtld.24.0059 39334547

[B163] Rifadin (2023). U. S. Food Drug Adm. (FDA). Available online at: https://www.accessdata.fda.gov/drugsatfda_docs/label/2023/050420s089,050627s034lbl.pdf.

[B164] Sarathy J. Blanc L. Alvarez-Cabrera N. Brien P. Dias-Freedman I. Mina M. (2019). Fluoroquinolone efficacy against tuberculosis is driven by penetration into lesions and activity against resident bacterial populations. Antimicrob. Agents Chemother. 63 (5), e02516-18–18. 10.1128/AAC.02516-18 30803965 PMC6496041

[B165] Sasahara K. Shimokawa Y. Hirao Y. Koyama N. Kitano K. Shibata M. (2015). Pharmacokinetics and metabolism of delamanid, a novel anti-tuberculosis drug, in animals and humans: importance of albumin metabolism *in vivo* . Drug Metabolism Dispos. 43, 1267–1276. 10.1124/dmd.115.064527 26055620

[B166] Schneider N. K. Thomas E. N. (2007). Addressing smoking cessation in tuberculosis control. Bull. World Health Organ. 85 (10), 820–821. 10.2471/blt.07.034797 18038065 PMC2636489

[B167] Seree E. M. Villard P. H. Re J. L. De Meo M. Lacarelle B. Attolini L. (1996). High inducibility of mouse renal CYP2E1 gene by tobacco smoke and its possible effect on DNA single strand breaks. Biochem. Biophysical Res. Commun. 219 (2), 429–434. 10.1006/bbrc.1996.0250 8605004

[B168] Serio A. W. Keepers T. Andrews L. Krause K. M. (2018). Aminoglycoside revival: review of a historically important class of antimicrobials undergoing rejuvenation. EcoSal Plus 8 (1). 10.1128/ecosalplus.ESP-0002-2018 PMC11575671 30447062

[B169] Sharma S. K. Mohan A. Singh A. D. Mishra H. Jhanjee S. Pandey R. M. (2018). Impact of nicotine replacement therapy as an adjunct to anti-tuberculosis treatment and behaviour change counselling in newly diagnosed pulmonary tuberculosis patients: an open-label, randomised controlled trial. Sci. Rep. 8 (1), 8828. 10.1038/s41598-018-26990-5 29891957 PMC5995820

[B170] Shi W. Zhang X. Jiang X. Yuan H. Lee J. S. Barry C. E. (2011). Pyrazinamide inhibits trans-translation in Mycobacterium tuberculosis. Sci. (New York, N.Y.) 333 (6049), 1630–1632. 10.1126/science.1208813 PMC3502614 21835980

[B171] Shih T.-Y. Pai C.-Y Yang P. Chang W.-L. Wang N.-C. Hu O. (2013). A novel mechanism underlies the hepatotoxicity of pyrazinamide. Antimicrob. Agents Chemother. 57 (4), 1685–1690. 10.1128/AAC.01866-12 23357778 PMC3623344

[B172] Shimada T. Yamazaki H. Mimura M. Inui Y. Guengerich F. P. (1994). Interindividual variations in human liver cytochrome P-450 enzymes involved in the oxidation of drugs, carcinogens and toxic chemicals: studies with liver microsomes of 30 Japanese and 30 caucasians. J. Pharmacol. Exp. Ther. 270 (1), 414–423. 10.1016/s0022-3565(25)22379-5 8035341

[B173] Shimokawa Y. Yoda N. Kondo S. Yamamura Y. Takiguchi Y. Umehara K. (2015). Inhibitory potential of twenty five anti-tuberculosis drugs on CYP activities in human liver microsomes. Biol. and Pharm. Bull. 38 (9), 1425–1429. 10.1248/bpb.b15-00313 26094899

[B174] Shinabarger D. L. Marotti K. R. Murray R. W. Lin A. H. Melchior E. P. Swaney S. M. (1997). Mechanism of action of oxazolidinones: effects of linezolid and eperezolid on translation reactions. Antimicrob. Agents Chemother. 41 (10), 2132–2136. 10.1128/aac.41.10.2132 9333037 PMC164082

[B175] Singh R. Manjunatha U. Boshoff H. I. M. Ha Y. H. Niyomrattanakit P. Ledwidge R. (2008). PA-824 kills nonreplicating Mycobacterium tuberculosis by intracellular NO Release, New York, N.Y. Science 322, 1392–1395. 10.1126/science.1164571 19039139 PMC2723733

[B176] Sirturo (2012). U. S. Food Drug Adm. (FDA). Available online at: https://www.accessdata.fda.gov/drugsatfda_docs/label/2012/204384s000lbl.pdf.

[B177] Slama K. Chiang C.-Y. Enarson D. A. Hassmiller K. Fanning A. Gupta P. (2007). Tobacco and tuberculosis: a qualitative systematic review and meta-analysis. Int. J. Tuberc. Lung Dis. Official J. Int. Union Against Tuberc. Lung Dis. 11 (10), 1049–1061. 17945060

[B178] Slatter J. G. Stalker D. J. Feenstra K. L. Welshman I. R. Bruss J. B. Sams J. P. (2001). Pharmacokinetics, metabolism, and excretion of linezolid following an oral dose of [(14)C]linezolid to healthy human subjects. Drug Metabolism Dispos. Biol. Fate Chem. 29 (8), 1136–1145. 11454733

[B179] Smit Z. van R. N. Binder A. Meldau R. Semple P. L. Evans A. (2014). Cigarette smoke impairs cytokine responses and BCG containment in alveolar macrophages. Thorax 69 (4), 363–370. 10.1136/thoraxjnl-2013-204229 24287167 PMC5523928

[B180] Smit Z. van R. N. Pai M. Yew W. W. Leung C. C. Zumla A. (2010). Global lung health: the colliding epidemics of tuberculosis, tobacco smoking, HIV and COPD. Eur. Respir. J. 35 (1), 27–33. 10.1183/09031936.00072909 20044459 PMC5454527

[B181] Song M. Zhang M. Han J. Fu W. (2024). Construction and validation of a nomogram to identify the risk of cavitation in pulmonary tuberculosis. Infect. Drug Resist. 17, 2803–2813. 10.2147/IDR.S459330 38989008 PMC11233379

[B182] Sopori M. (2002). Effects of cigarette smoke on the immune system. Nat. Rev. Immunol. 2 (5), 372–377. 10.1038/nri803 12033743

[B183] Stepanov I. Villalta P. W. Knezevich A. Jensen J. Hatsukami D. Hecht S. S. (2010). Analysis of 23 polycyclic aromatic hydrocarbons in smokeless tobacco by gas chromatography-mass spectrometry. Chem. Res. Toxicol. 23 (1), 66–73. 10.1021/tx900281u 19860436 PMC2807893

[B184] Strydom N. Gupta S. V. Fox W. S. Via L. E. Bang H. Lee M. (2019). Tuberculosis drugs’ distribution and emergence of resistance in patient’s lung lesions: a mechanistic model and tool for regimen and dose optimization. PLoS Med. 16 (4), e1002773. 10.1371/journal.pmed.1002773 30939136 PMC6445413

[B185] Sullivan O. Poitevin F. Sierra R. G. Gati C. Han Dao E. Rao Y. (2018). Aminoglycoside ribosome interactions reveal novel conformational States at ambient temperature. Nucleic Acids Res. 46 (18), 9793–9804. 10.1093/nar/gky693 30113694 PMC6182148

[B186] Sumida A. Fukuen S. Yamamoto I. Matsuda H. Naohara M. Azuma J. (2000). Quantitative analysis of constitutive and inducible CYPs mRNA expression in the HepG2 cell line using reverse transcription-competitive PCR. Biochem. Biophysical Res. Commun. 267 (3), 756–760. 10.1006/bbrc.1999.2029 10673364

[B187] Szumowski J. D. Lynch J. B. (2015). Profile of delamanid for the treatment of multidrug-resistant tuberculosis. Drug Des. Dev. Ther. 9, 677–682. 10.2147/DDDT.S60923 PMC4319680 25678771

[B188] Tanoglu A. Erdem H. Friedland J. S. Ankaralı H. Garcia-Goez J. F. Albayrak A. (2023). Clinicopathological profile of peritoneal tuberculosis and a new scoring model for predicting mortality: an international ID-IRI study. Eur. J. Clin. Microbiol. and Infect. Dis. 42 (8), 981–992. *Official Publication of the European Society of Clinical Microbiology* . 10.1007/s10096-023-04630-9 37318601

[B189] Tantcheva-Poór I. Zaigler M. Rietbrock S. Fuhr U. (1999). Estimation of cytochrome P-450 CYP1A2 activity in 863 healthy caucasians using a saliva-based caffeine test. Pharmacogenetics 9 (2), 131–144. 10376760

[B190] Tatro D. S. (2014). Drug interaction Facts 2015. Lippincott Williams and Wilkins.

[B191] TB Disease Burden (2024). TB disease burden. Available online at: https://www.who.int/teams/global-tuberculosis-programme/tb-reports/global-tuberculosis-report-2023/tb-disease-burden (Accessed November 29, 2024).

[B192] TB Incidence (2024). TB incidence. Available online at: https://www.who.int/teams/global-tuberculosis-programme/tb-reports/global-tuberculosis-report-2023/tb-disease-burden/1-1-tb-incidence (Accessed November 29, 2024).

[B193] TB Preventive Treatment (2025). Identifying populations for TB preventive treatment TB knowledge sharing. Available online at: https://tbksp.who.int/en/node/631 (Accessed June 19, 2025).

[B194] The End TB Strategy (2025). The End TB strategy. Available online at: https://www.who.int/publications/i/item/WHO-HTM-TB-2015.19 (Accessed June 19, 2025).

[B195] Tilley A. E. Walters M. S. Shaykhiev R. Crystal R. G. (2015). Cilia dysfunction in lung disease. Annu. Rev. Physiology 77, 379–406. 10.1146/annurev-physiol-021014-071931 PMC4465242 25386990

[B196] Tobacco Collaborators (2017). Smoking prevalence and attributable disease burden in 195 countries and territories, 1990-2015: a systematic analysis from the global burden of disease study 2015. Lancet London, Engl. 389 (10082), 1885–1906. 10.1016/S0140-6736(17)30819-X PMC5439023 28390697

[B197] Tobacco Collaborators (2021). Spatial, temporal, and demographic patterns in prevalence of smoking tobacco use and attributable disease burden in 204 countries and territories, 1990-2019: a systematic analysis from the global burden of disease study 2019. Lancet London, Engl. 397 (10292), 2337–2360. 10.1016/S0140-6736(21)01169-7 PMC8223261 34051883

[B198] Tostmann A. Boeree M. J. Aarnoutse R. E. de Lange W. C. M. van der Ven A. J. A. M. Dekhuijzen R. (2008). Antituberculosis drug-induced hepatotoxicity: concise up-to-Date review. J. Gastroenterology Hepatology 23 (2), 192–202. 10.1111/j.1440-1746.2007.05207.x 17995946

[B199] Trecator (2016). U. S. Food Drug Adm. (FDA). Available online at: https://www.accessdata.fda.gov/drugsatfda_docs/label/2016/013026s029lbl.pdf.

[B200] Tuberculosis and HIV (2025). Tuberculosis and HIV. Available online at: https://www.who.int/teams/global-hiv-hepatitis-and-stis-programmes/hiv/treatment/tuberculosis-hiv (Accessed June 19, 2025).

[B201] Unissa A. N. Subbian S. Hanna L. E. Selvakumar N. (2016). Overview on mechanisms of isoniazid action and resistance in Mycobacterium tuberculosis. Infect. Genet. Evol. J. Mol. Epidemiol. Evol. Genet. Infect. Dis. 45, 474–492. 10.1016/j.meegid.2016.09.004 27612406

[B202] Urakami Y. Okuda M. Masuda S. Saito H. Inui K. I. (1998). Functional Characteristics and membrane localization of rat multispecific organic cation transporters, OCT1 and OCT2, mediating tubular secretion of cationic drugs. J. Pharmacol. Exp. Ther. 287 (2), 800–805. 10.1016/s0022-3565(24)37859-0 9808712

[B203] Vanino E. Granozzi B. Akkerman O. W. Munoz-Torrico M. Palmieri F. Seaworth B. (2023). Update of drug-resistant tuberculosis treatment guidelines: a turning point. Int. J. Infect. Dis. IJID Official Publ. Int. Soc. Infect. Dis. 130 (Suppl. 1), S12–S15. 10.1016/j.ijid.2023.03.013 36918080

[B204] Villard P.-H. Hcrber R. Sérée E. M. Attolini L. Magdalou J. Bruno L. (1998). Effect of cigarette smoke on UDP-glucuronosyltransferase activity and cytochrome P450 content in liver, lung and kidney microsomes in mice. Pharmacol. and Toxicol. 82 (2), 74–79. 10.1111/j.1600-0773.1998.tb01401.x 9498235

[B205] Vistisen K. Loft S. Poulsen H. E. (1991). Cytochrome P450 IA2 activity in man measured by caffeine metabolism: effect of smoking, broccoli and exercise. Adv. Exp. Med. Biol. 283, 407–411. 10.1007/978-1-4684-5877-0_55 2069014

[B206] Voutsinas J. Wilkens L. R. Adrian F. Vogt T. M. Yokochi L. A. Decker R. (2013). Heterocyclic amine intake, smoking, cytochrome P450 1A2 and N-acetylation phenotypes, and risk of colorectal adenoma in a multiethnic population. Gut 62 (3), 416–422. 10.1136/gutjnl-2011-300665 22628494 PMC4491437

[B207] Wallace B. J. Tai P. C. Herzog E. L. Davis B. D. (1973). Partial inhibition of polysomal ribosomes of Escherichia coli by streptomycin. Proc. Natl. Acad. Sci. U. S. A. 70 (4), 1234–1237. 10.1073/pnas.70.4.1234 4577795 PMC433465

[B208] Wang E. Y. Arrazola R. A. Mathema B. Ahluwalia I. B. Mase S. R. (2020). The impact of smoking on tuberculosis treatment outcomes: a meta-analysis. Int. J. Tuberc. Lung Dis. Official J. Int. Union Against Tuberc. Lung Dis. 24 (2), 170–175. 10.5588/ijtld.19.0002 PMC7232866 32127100

[B209] Wang H.-T. Lin J.-H. Yang C.-H. Haung C.-H. Weng C.-W. Lin A.M.-Y. (2017). Acrolein induces mtDNA damages, mitochondrial fission and mitophagy in human lung cells. Oncotarget 8 (41), 70406–70421. 10.18632/oncotarget.19710 29050289 PMC5642564

[B210] Wang L. Ma Y. Duan H. Yao J. Liang Li Zhang R. (2015). Pharmacokinetics and tissue distribution study of PA-824 in rats by LC-MS/MS. J. Chromatogr. B, Anal. Technol. Biomed. Life Sci. 1006, 194–200. 10.1016/j.jchromb.2015.10.039 26554313

[B211] Wang P. Pradhan K. Zhong X. Ma X. (2016). Isoniazid metabolism and hepatotoxicity. Acta Pharm. Sin. B 6 (5), 384–392. 10.1016/j.apsb.2016.07.014 27709007 PMC5045547

[B212] Waters M. Tadi P. (2024). “Streptomycin,” in *StatPearls*. Treasure island (FL) (StatPearls Publishing). Available online at: http://www.ncbi.nlm.nih.gov/books/NBK555886/.

[B213] WHO Consolidated Guidelines on Tuberculosis (2024). WHO consolidated guidelines on tuberculosis: Module 4: treatment: drug-resistant tuberculosis treatment. Available online at: https://www.who.int/publications/i/item/9789240007048 (Accessed November 25, 2024). 32603040

[B214] WHO Consolidated Guidelines on Tuberculosis Module 1 (2024). WHO consolidated guidelines on tuberculosis Module 1: prevention - tuberculosis preventive treatment, Second Edition. Available online at: https://www.who.int/publications/i/item/9789240096196 (Accessed November 29, 2024). 39298638

[B215] WHO Global (2024). Report on trends in prevalence of tobacco use 2000-2025, Fourth Edition. Available online at: https://www.who.int/publications/i/item/9789240039322 (Accessed November 25, 2024).

[B216] WHO Global (2025). Report on trends in prevalence of tobacco use 2000–2030. Available online at: https://www.who.int/publications/i/item/9789240088283 (Accessed June 19, 2025).

[B217] WHO Report on the Global Tobacco Epidemic (2019). Offer help to quit tobacco use. Available online at: https://www.who.int/publications/i/item/9789241516204 (Accessed June 19, 2025).

[B218] WHO Report on the Global Tobacco Epidemic (2023). Protect people from tobacco smoke. Available online at: https://www.who.int/publications/i/item/9789240077164 (Accessed November 25, 2024).

[B219] Yano T. Kassovska-Bratinova S. Shin Teh J. Winkler J. Sullivan K. Isaacs A. (2011). Reduction of clofazimine by mycobacterial type 2 NADH:quinone oxidoreductase: a pathway for the generation of bactericidal levels of reactive oxygen species. J. Biol. Chem. 286 (12), 10276–10287. 10.1074/jbc.M110.200501 21193400 PMC3060482

[B220] Yen Y.-F. Yen M.-Y. Lin Y.-S. Lin Y.-P. Shih H.-C. Li L.-H. (2014). Smoking increases risk of recurrence after successful anti-tuberculosis treatment: a population-based study. Int. J. Tuberc. Lung Dis. Official J. Int. Union Against Tuberc. Lung Dis. 18 (4), 492–498. 10.5588/ijtld.13.0694 24670708

[B221] Ying L. Zhu H. Shoji S. Fredrick K. (2019). Roles of specific aminoglycoside-ribosome interactions in the inhibition of translation. RNA (New York, N.Y.) 25 (2), 247–254. 10.1261/rna.068460.118 30413565 PMC6348987

[B222] Yue J. Peng R. Yang J. Kong R. Liu J. (2004). CYP2E1 mediated isoniazid-induced hepatotoxicity in rats. Acta Pharmacol. Sin. 25 (5), 699–704. 15132840

[B223] Zaverucha-do-Valle C. Monteiro S. P. El-Jaick K. B. Rosadas L. A. Costa M. J. M. Quintana M. S. B. (2014). The role of cigarette smoking and liver enzymes polymorphisms in anti-tuberculosis drug-induced hepatotoxicity in Brazilian patients. Tuberc. Edinb. Scotl. 94 (3), 299–305. 10.1016/j.tube.2014.03.006 24793319

[B224] Zevin S. Benowitz N. L. (1999). Drug interactions with tobacco smoking. An update. Clin. Pharmacokinet. 36 (6), 425–438. 10.2165/00003088-199936060-00004 10427467

[B225] Zhang C. Ou A. Jin L. Yang N. Deng P. Guan C. (2024). Cadmium exposure triggers alveolar epithelial cell pyroptosis by inducing mitochondrial oxidative stress and activating the cGAS-STING pathway. Cell. Commun. Signal. CCS 22 (1), 566. 10.1186/s12964-024-01946-7 39587603 PMC11590492

[B226] Zhang L. Zhao Y. Gao Y. Wu L. Gao R. Qi Z. (2020). Structures of cell wall arabinosyltransferases with the anti-tuberculosis drug ethambutol. Sci. (New York, N.Y.) 368 (6496), 1211–1219. 10.1126/science.aba9102 32327601

[B227] Zhao Q. Chen Y. Huang W. Zhou H. Zhang W. (2023). Drug-microbiota interactions: an emerging priority for precision medicine. Signal Transduct. Target. Ther. 8 (1), 386. 10.1038/s41392-023-01619-w 37806986 PMC10560686

[B228] Zimhony O. Vilchèze C. Arai M. Welch J. T. Jacobs W. R. (2007). Pyrazinoic acid and its N-propyl ester inhibit fatty acid synthase type I in replicating tubercle bacilli. Antimicrob. Agents Chemother. 51 (2), 752–754. 10.1128/AAC.01369-06 17101678 PMC1797748

[B229] Zumla A. Sahu S. Ditiu L. Singh U. Park Y.-J. Yeboah-Manu D. (2025). Inequities underlie the alarming resurgence of tuberculosis as the world’s top cause of death from an infectious disease - breaking the silence and addressing the underlying root causes. IJID Reg. 14 (Suppl. 2), 100587. 10.1016/j.ijregi.2025.100587 40201557 PMC11973691

[B230] Zyvox (2014). U. S. Food Drug Adm. (FDA). Available online at: https://www.accessdata.fda.gov/drugsatfda_docs/label/2014/021130s032,021131s026,021132s031lbl.pdf.

