# Supplementary material for: A scoping review about smoking, smoking cessation and their effects on anti-tuberculosis agents: insights into drug metabolisms, safety, and effectiveness
Source: Front Pharmacol. 2025 Jul 16;16:1606150. doi: 10.3389/fphar.2025.1606150 (PMC12308143; doi:10.3389/fphar.2025.1606150)
Supplement: Supplementary file 1 [file Table1.docx]

Supplementary Material

# Supplementary Table

**Table**: Search string details adopted for each database.

| **Database** | |
| --- | --- |
| **MEDLINE (PubMed)** | **Embase** |
| (tuberculosis [MeSH Terms]) OR (tuberculosis [Title/Abstract]) OR (TB [Title/Abstract]) OR (pulmonary tuberculosis [Title/Abstract]) OR (pulmonary TB [Title/Abstract]) AND (smoking [MeSH Terms]) OR (smoking [Title/Abstract]) OR (smoke [Title/Abstract]) OR (cigarette [Title/Abstract]) OR (tobacco [MeSH Terms]) OR (tobacco products [MeSH Terms]) OR (tobacco [Title/Abstract]) AND (antituberculous drugs [Title/Abstract]) OR (anti-TB agents [MeSH Terms]) OR (antituberculous medications [Title/Abstract]) OR (rifampicin [MeSH Terms]) OR (rifampicin [Title/Abstract]) OR (rifampin [Title/Abstract]) OR (rifampin [MeSH Terms]) OR (isoniazid [Title/Abstract]) OR (isoniazid [MeSH Terms]) OR (pyrazinamide [Title/Abstract]) OR (pyrazinamide [MeSH Terms]) OR (ethambutol [Title/Abstract]) OR (ethambutol [MeSH Terms]) OR (rifabutin [Title/Abstract]) OR (rifabutin [MeSH Terms]) OR (rifapentine [Title/Abstract]) OR (rifapentine [MeSH Terms]) OR (streptomycin [Title/Abstract]) OR (streptomycin [MeSH Terms]) OR (capreomycin [Title/Abstract]) OR (capreomycin [MeSH Terms]) OR (amikacin [Title/Abstract]) OR (amikacin [MeSH Terms]) OR (levofloxacin [Title/Abstract]) OR (levofloxacin [MeSH Terms]) OR (moxifloxacin [Title/Abstract]) OR (moxifloxacin [MeSH Terms]) OR (linezolid [Title/Abstract]) OR (linezolid [MeSH Terms]) OR (ethionamide [Title/Abstract]) OR (ethionamide [MeSH Terms]) OR (clofazimine [Title/Abstract]) OR (clofazimine [MeSH Terms]) OR (bedaquiline [Title/Abstract]) OR (bedaquiline [MeSH Terms]) OR (pretomanid [Title/Abstract]) OR (pretomanid [MeSH Terms]) OR (delamanid [Title/Abstract]) OR (delamanid [MeSH Terms]) AND (nicotine replacement therapies [Title/Abstract]) OR (nicotine replacement therapies [MeSH Terms]) OR (nicotine replacement products [Title/Abstract]) OR (nicotine replacement products [MeSH Terms])) OR (NRT [Title/Abstract])) OR (NRT [MeSH Terms]) OR (NRTs [Title/Abstract]) OR (NRTs [MeSH Terms]) OR (varenicline [Title/Abstract]) OR (varenicline [MeSH Terms]) OR (cytisine [Title/Abstract]) OR (cytisine [MeSH Terms]) OR (bupropion [Title/Abstract]) OR (bupropion [MeSH Terms]) AND (cytochrome P450 [Title/Abstract]) OR (cytochrome P450 [MeSH Terms]) OR (cytochrome p450 enzyme system [Title/Abstract]) OR (cytochrome p450 enzyme system [MeSH Terms]) OR (CYP [Title/Abstract]) OR (CYP [MeSH Terms]) OR (uridine5'-diphospho-glucuronosyltransferase [Title/Abstract]) OR (uridine5'-diphospho-glucuronosyltransferase [MeSH Terms]) OR (UGT [Title/Abstract]) OR (UGT [MeSH Terms]) | #1 'tuberculosis'/exp OR 'pulmonary tuberculosis'/exp  #2 'smoke'/exp OR 'smoking'/exp OR 'smokers'/exp OR 'tobacco'/exp OR 'cigarette'/exp  #3 'antituberculous drugs'/exp OR 'anti-TB medications'/exp OR 'rifampicin'/exp OR 'isoniazid'/exp OR 'pyrazinamide'/exp OR 'ethambutol'/exp OR 'rifabutin'/exp OR 'rifapentine'/exp OR 'streptomycin'/exp OR 'capreomycin'/exp OR 'amikacin'/exp OR 'levofloxacin'/exp OR 'moxifloxacin'/exp OR 'linezolid'/exp OR 'ethionamide'/exp OR 'clofazimine'/exp OR 'bedaquiline'/exp OR 'pretomanid'/exp OR 'delamanid'/exp  #4 'Cytochrome P450'/exp OR 'CYP'/exp OR 'Uridine 5'-diphospho-glucuronosyltransferase'/exp OR 'UGT'/exp  #1 AND #2 AND #3 AND #4 |
